# Quantitative proteomic analysis of pancreatic cyst fluid proteins associated with malignancy in intraductal papillary mucinous neoplasms

**DOI:** 10.1186/s12014-018-9193-1

**Published:** 2018-04-18

**Authors:** Misol Do, Dohyun Han, Joseph Injae Wang, Hyunsoo Kim, Wooil Kwon, Youngmin Han, Jin-Young Jang, Youngsoo Kim

**Affiliations:** 1 Department of Biomedical Sciences, Seoul National University College of Medicine, 28 Yeongeon-dong, Seoul, 110-799 Korea; 2 Department of Biomedical Engineering, Seoul National University College of Medicine, 28 Yeongeon-dong, Seoul, 110-799 Korea; 3 Department of Surgery, Seoul National University College of Medicine, 28 Yeongeon-dong, Seoul, 110-799 Korea; 4 Proteomics Core Facility, Biomedical Research Institute, Seoul National University Hospital, 101 Daehak-ro, Seoul, Korea

**Keywords:** Pancreatic cyst fluid, Intraductal papillary mucinous neoplasm (IPMN), IPMN dysplasia, Biomarker candidates, LC–MS/MS

## Abstract

**Background:**

The application of advanced imaging technologies for identifying pancreatic cysts has become widespread. However, accurately differentiating between low-grade dysplasia (LGD), high-grade dysplasia (HGD), and invasive intraductal papillary mucinous neoplasms (IPMNs) remains a diagnostic challenge with current biomarkers, necessitating the development of novel biomarkers that can distinguish IPMN malignancy.

**Methods:**

Cyst fluid samples were collected from nine IPMN patients (3 LGD, 3 HGD, and 3 invasive IPMN) during their pancreatectomies. An integrated proteomics approach that combines filter-aided sample preparation, stage tip-based high-pH fractionation, and high-resolution MS was applied to acquire in-depth proteomic data of pancreatic cyst fluid and discover marker candidates for IPMN malignancy. Biological processes of differentially expressed proteins that are related to pancreatic cysts and aggressive malignancy were analyzed using bioinformatics tools such as gene ontology analysis and Ingenuity pathway analysis. In order to confirm the validity of the marker candidates, 19 cyst fluid samples were analyzed by western blot.

**Results:**

A dataset of 2992 proteins was constructed from pancreatic cyst fluid samples. A subsequent analysis found 2963 identified proteins in individual samples, 2837 of which were quantifiable. Differentially expressed proteins between histological grades of IPMN were associated with pancreatic diseases and malignancy according to ingenuity pathway analysis. Eighteen biomarker candidates that were differentially expressed across IPMN histological grades were discovered—7 DEPs that were upregulated and 11 that were downregulated in more malignant grades. HOOK1 and PTPN6 were validated by western blot in an independent cohort, the results of which were consistent with our proteomic data.

**Conclusions:**

This study demonstrates that novel biomarker candidates for IPMN malignancy can be discovered through proteomic analysis of pancreatic cyst fluid.

**Electronic supplementary material:**

The online version of this article (10.1186/s12014-018-9193-1) contains supplementary material, which is available to authorized users.

## Background

Intraductal papillary mucinous neoplasms (IPMNs) are precancerous lesions that grow in the pancreatic ducts and are characterized by papillary growth of the ductal epithelium. The production of thick mucinous fluid, another hallmark of IPMNs, causes cystic dilation and can progress into pancreatic ductal adenocarcinoma [1–4]. Depending on the malignancy, IPMN is classified as low-grade dysplasia (LGD), intermediate-grade dysplasia (IGD), high-grade dysplasia (HGD), and invasive IPMN. According to the official guidelines for managing pancreatic IPMN, only patients with HGD or invasive IPMN require surgery, because they are at higher risk of their disease developing into cancer [5]. Milder forms of IPMN can be managed with active surveillance and do not warrant surgical intervention. However, current methods for assessing the histological grades of IPMNs are unreliable, and as a result, patients with milder IPMN are often subjected to unnecessary operations [6–10].

In clinical practice, MRI and CT scans, cytological examination of cyst fluid, measurement of tumor markers such as carcinoembryonic antigen (CEA) and carbohydrate antigen 19-9 (CA 19-9), and analysis of GTPase Kras (KRAS) and guanine nucleotide-binding protein alpha subunit (GNAS) mutations are used to categorize patients with pancreatic cysts [6, 7, 10–16]. Features of pancreatic images in MRI or CT scans are generally used to assess the potential malignancy of cysts but have low diagnostic accuracy—up to 40% of neoplastic cysts are misdiagnosed as pseudocysts, and the overall accuracy ranges from 20 to 80% [17–19]. Cytological examination of pancreatic cyst fluid is an alternative approach, but it has difficulties in identifying the existence of malignancy when sufficient sample volumes are unavailable [16, 20–23]. Differentiating mucinous cysts from other cystic lesions by measuring carcinoembryonic antigen levels in cyst fluid has relatively low accuracy (79% sensitivity, 73% specificity) [17, 24]. Similarly, as shown by Frossard et al. [25], CA 19-9, a pancreatic cancer marker, also performs poorly in distinguishing mucinous cysts and other lesions, with 15% sensitivity and 81% specificity [16]. Analyzing GNAS mutations are only applicable for samples that are acquired during the early stages of IPMN [20, 23, 26, 27]. The general consensus is that existing methods for diagnosing IPMN histological grades are imprecise and unreliable, even when used in tandem [6, 7, 10, 16, 17, 20].

Because pancreatic cyst fluid contains secreted proteins from tumor cells at higher proportions, several groups, such as Poersch et al. [28], have concluded that it is a better experimental model of IPMN histological grades than serum and plasma [16, 29–32]. Consequently, pancreatic cyst fluid has been widely favored in recent research on IPMN, because it is obtainable by endoscopic ultrasound-guided fine needle aspiration biopsy, which is minimally invasive [6, 25, 33]. Many studies have focused on discovering protein markers that differentiate mucinous from nonmucinous cyst fluid and cyst fluid that is related to IPMN dysplasia, based on DNA methylation and telomerase activity, as demonstrated by Hata et al. [20]. Diagnosing histological grades of IPMN using pancreatic cyst fluid by proteomic analysis is a relatively unexplored area [6, 7, 20, 34, 35]. Thus, the IPMN dysplasia proteome has not been characterized extensively.

Cuoghi et al. [36] performed a cursory profiling study of the proteomic patterns of pancreatic cyst fluids from various cystic lesions, including IPMN, MCN, serous cystadenomas, pancreatic neuroendocrine tumors, and pseudocysts, identifying 220–727 proteins in these fluids. Specifically, 243 proteins were identified in the IPMN groups. Gbormittah et al. [37] characterized glycoproteins and nonglycoproteins in mucinous and nonmucinous pancreatic cyst fluid to identify DEPs as potential biomarker targets. They found 230 proteins in mucinous subtypes and 290 proteins in nonmucinous subtypes; the DEPs between mucinous and nonmucinous cyst fluid were associated with lipid metabolism, energy metabolism, and stress responses. These studies were unable to determine the IPMN histological grades, merely differentiating between mucinous and nonmucinous cyst fluid. These recent studies demonstrate that the current cyst fluid proteome lacks the coverage to extrapolate meaningful conclusions on the molecular and biological activities of the identified proteins, which ultimately impedes our understanding of IPMN histology in terms of proteomic differences and biological functions.

In this report, we aimed to comprehensively identify pancreatic cyst fluid proteins and discover differentially expressed proteins in accordance with histological grades of IPMN. Recently, we reported a platform for in-depth profiling of pancreatic cyst fluid [38]. Using this platform, the protein expression patterns of pancreatic cyst fluid were analyzed on a high-resolution mass spectrometer to discover potential biomarkers of IPMN histological grades. Subsequently, we validated some of the 18 candidate markers by western blot. We report here that pancreatic cyst fluid is a valuable source for biomarker studies as it contains putative markers related to IPMNs and that bioinformatics analyses using identified proteins of cyst fluid enhance our understanding of IPMNs at the molecular level. We ultimately intend to discover marker candidates that can help patients avoid unnecessary operations.

## Methods

### Clinical samples

Cyst fluid samples were collected from 9 IPMN patients during their pancreatectomies at Seoul National University Hospital (Seoul, South Korea) from April 2013 to December 2015. At least 200 μL of cyst fluid was aspirated from each patient. The samples were then snap-frozen in liquid nitrogen and stored at − 80 °C. All patients consented to participation in the study in accordance with Institutional Review Board guidelines (IRB No. 1301-095-458). IPMN samples were divided into low-grade dysplasia (LGD, n = 3), high-grade dysplasia (HGD, n = 3), and invasive IPMN (n = 3).

### Pancreatic cyst fluid protein sample preparation

Each pancreatic cyst fluid sample was transferred to an Eppendorf tube. Viscous samples that could not be pipetted were sonicated briefly (Sonics and Materials Inc., USA) to remove the mucus. All samples were centrifuged at 15,000 rpm for 20 min at 4 °C, and the supernatant was placed into a new tube. The protein concentration was estimated using a BCA reducing agent compatibility assay kit (Thermo Scientific, Rockford, IL, USA). Equal portions of each sample were pooled to create a peptide library from 600 µg of proteins. One hundred micrograms of individual protein samples were used for label-free quantification. Cold acetone (Sigma-Aldrich, USA) was added to the supernatant to the ratio of 5:1 (v/v) to precipitate the proteins. The mixture was vortexed gently and incubated overnight at − 20 °C. The precipitate was centrifuged for 10 min (15,000 rpm at 4 °C), and the supernatant was carefully decanted, after which 500 µL cold acetone was added to the pellet. After this wash step, the pellet was centrifuged for 10 min (15,000 rpm at 4 °C). The remaining acetone was poured off, and the pellet was air-dried for 2 h.

### Protein digestion and desalting

The pellet was dissolved in 30 μL of lysis buffer (4% SDS, 0.1 M DTT, 0.1 M Tris–Cl, pH 7.4). The mixture was gently vortexed and boiled for 30 min at 95 °C. The boiled mixture was then transferred through a 30-kDa cutoff filter (Amicon^®^ Ultra, Millipore, USA) with 300 μL 8 M urea (8 M Urea, 0.1 M Tris–Cl, pH 8.5) and centrifuged (14,000*g*, 15 min, 20 °C). This filtration step was repeated twice to dilute and lower the SDS concentration. Next, 200 μL 50 mM IAA (50 mM IAA, 8 M urea, 0.1 M Tris–Cl, pH 8.5) was added to each sample and incubated for 1 h at 25 °C. Each sample was then centrifuged and washed twice with 300 μL 8 M urea and then three times with 300 μL 40 mM ammonium bicarbonate (ABC).

After the samples were centrifuged, 100 μL 40 mM ABC and 0.1 μg/μL trypsin (at a trypsin:sample ratio of 1:80, wt/wt) were added to each sample and incubated for 18 h at 37 °C. Next, the filters (nine individual samples, one pooled sample) were transferred to new collection tubes, which were centrifuged after 100 μL 40 mM ABC was added. Fifty microliters NaCl was added to each individual sample, and 50 μL water was added to the pooled sample. The pooled sample underwent an additional digestion step [39, 40]. Again, the filter unit was transferred to a new tube and centrifuged after 200 μL 8 M urea was added. Then, the unit was centrifuged twice with 300 μL 40 mM ABC. One-tenth of the concentration of trypsin that was used in the first digestion step was added with 100 μL 40 mM ABC, and the unit was incubated for 18 h at 37 °C. Next, the filter was transferred to another tube, and the peptides were collected by sequential centrifugation with 100 μL 40 mM ABC and 50 μL 0.5 M NaCl.

Prior to acidification and desalting, all tryptic peptides were measured by tryptophan fluorescence assay to determine the volume that was required to extract the same amount of peptides from each sample [41]. The equalized amounts of peptides were then set aside for label-free quantification. The measured peptides were acidified with 10 μL 10% TFA and desalted with homemade C18-StageTip columns as described [42]. The desalted peptides were then lyophilized on a speed-vacuum centrifuge and stored at − 80 °C.

### Peptide fractionation by high-pH reverse phase fractionation

To increase the number of identified proteins, the pooled cyst fluid sample was fractionated using two methods: modified stage-tip-based high-pH peptide fractionation [43, 44] and offline HPLC high-pH fractionation on an Agilent 1260 Bio-inert. For stage-tip fractionation, half of the lyophilized peptides were dissolved in 200 μL of loading buffer (15 mM ammonium hydroxide solution, pH 10, and 2% acetonitrile) and separated on a pipette-based C18 RP microcolumn. The column was constructed by plugging the bottom of a 200 μL transparent pipette tip with C18 Empore disk membrane (3 M, Bracknell, UK) and packing the tip with POROS 20 R2 resin. The plugged tip was rinsed three times with 100 μL 100% methanol and then three times with 100 μL 100% acetonitrile (ACN). The column was then conditioned with 100 μL of loading buffer using a syringe. The peptides were loaded onto the column at pH 10. An ACN gradient of 2, 5, 7.5, 10, 12.5, 15, 17.5, 20, 22.5, 25, 27.5, 30, 32.5, 35, 40, 50, 60, 70, 80, and 100% was used to elute 20 fractions, which were collected into six tubes discontinuously to distribute eluents of varying hydrophobicity. These six fractions were lyophilized in a speed-vacuum centrifuge and stored at − 80 °C.

The remaining half of the lyophilized peptides was dissolved in 80 μL of loading buffer (15 mM ammonium hydroxide in water, pH 10). The peptides were loaded onto the column, and 96 (2 mL Square Collection Plate, Waters, UK) fractions were eluted by applying an ACN gradient (pH 10, 5–35%) for 40 min at a flow rate of 0.2 mL/min and washing the column with 90% ACN for 10 min at 0.2 mL/min. The ACN gradient was established by mixing varying proportions of solution A (0.1% formic acid in HPLC-grade distilled water) and solution B (0.1% formic acid in ACN). The 96 fractions were concatenated according to the column number of the plate to produce 12 pooled fractions. The resulting 12 tubes were lyophilized in a speed-vacuum centrifuge and stored at − 80 °C.

### LC–MS/MS analysis

The peptide samples were analyzed using an LC–MS/MS configuration, comprising an Easy-nLC 1000 (Thermo Fisher Scientific, Waltham, MA, USA) that was coupled to a Q Exactive mass spectrometer with a nanoelectrospray ion source (Thermo Fisher Scientific, Waltham, MA, USA), per our established protocol [38, 44, 45]. Peptides were separated on a 2-column system that was composed of a trap column (75 μm I.D. × 2 cm, C18 3.0 μm, 100 Å) and an analytical column (50 μm I.D. × 15 cm, C18 3.0 μm, 100 Å).

Fractionated peptides were subjected to an ACN gradient (6–60%) for 235 min. The gradient was created by mixing solvent A (2% ACN and 0.1% v/v formic acid) and solvent B (100% acetonitrile and 0.1% v/v formic acid) at various proportions. The spray voltage was set to 2.0 kV in positive ion mode, and the temperature of the heated capillary was set to 320 °C. Mass spectra were acquired in data-dependent mode by top 20 method on an Orbitrap analyzer with a mass range of 350–1700 *m/z* and a resolution of 70,000 at *m/z* 200. HCD scans were acquired at a resolution of 17,500. HCD peptide fragments were acquired at a normalized collision energy (NCE) of 27. The maximum ion injection time for the survey scan and MS/MS scan was 20 and 80 ms, respectively. All samples were analyzed in three technical replicates.

### Raw data search

The MS data from the Q Exactive were processed in MaxQuant (version 1.5.5.1 with built-in Andromeda search engine) [46]. Precursor MS signal intensities were determined, and HCD MS/MS spectra were de-isotoped and filtered, such that only the 20 most abundant fragments per 100 *m/z* range were retained. Protein groups were identified by searching the MS and MS/MS data of the peptides against the Uniprot human database (2014 December, 88,717 entries). Both the forward and reverse amino acid sequences were taken into account when calculating the false discovery rate (FDR). Following established target-decoy search procedures [47], search results were filtered at FDR < 1% for identifying peptides, modification sites, and proteins. The search was conducted in digestion mode trypsin/P, which assumes cleavage at carboxyl sides of lysine and arginine, including cases where the subsequent residue is a proline.

The following parameters were used in the database search: precursor and HCD fragment mass tolerances of 6 and 20 ppm, respectively; tolerance of up to two missed cleavages; carbamidomethylation of cysteine as a fixed modification; and oxidation of Met and acetylation of protein N-term as variable modifications. The minimum peptide length was set to six residues. Peptides were assigned to protein groups by the principle of parsimony [48–50]. The principle is applied to derive the smallest list of probable protein groups that adequately represent the identified peptides, which reduces sequence redundancy issues. All proteomics data in this report have been deposited in the ProteomeXchange Consortium (http://proteomecentral.proteomexchange.org/) through the PRIDE partner repository: dataset identifier PXD008302 [51, 52].

### Label-free quantification and statistical analysis

Label-free quantification (LFQ) and statistical analysis were performed in MaxQuant (version 1.5.5.1) and Perseus (version 1.5.8.5), respectively, according to our previous studies [43, 45]. Protein abundance was obtained from LFQ intensity values. LFQ intensity was calculated as described by the equation by Cox et al. [53]. Each of the three histological groups in this study had three biological replicates, which in turn had three technical replicates each. Thus, a total of 9 LFQ intensity values exist per histological group (three biological replicates × three technical replicates). LFQ intensity values greater than zero were deemed valid. Proteins with at least six valid values within a histological group were used in statistical analysis for label-free quantification. This criterion was used to reduce the possibility of analyzing proteins that are nonspecific to histological grades. After log2-transformation of protein intensities, the missing values were replaced with expected intensities based on the normal distribution (imputation width = 0.3, shift = 1.8) of log2-transformed LFQ intensities [43]. Student’s *t* test was applied to the preprocessed dataset of matched proteins to detect DEPs across grades of IPMN dysplasia. The comparative pairs for the statistical analysis were LGD versus HGD (comparison 1), HGD versus invasive IPMN (comparison 2), and LGD versus invasive IPMN (comparison 3). A Benjamini–Hochberg FDR threshold of 0.05 was applied to each pair to find significantly changed proteins. Subsequently, the expression patterns of overlapping DEPs across two or more pairs were analyzed to screen for biomarker candidates. DEPs that had expression patterns that varied based on the malignancy of IPMN were selected as final biomarker candidates. The resulting DEPs were subjected to hierarchical clustering in Perseus (version 1.5.8.5) with the following parameters: Euclidean distance, average linkage, the number of clusters of 100, maximal number of iterations of 10, the number of restarts of 1, and k-means preprocessing prior to clustering.

### Bioinformatics analysis

The gene ontologies (GOs) of all DEPs were annotated using the DAVID bioinformatics resource tool (https://david.ncifcrf.gov/) and the UniprotKB database (http://www.uniprot.org/). The GO analysis included information on biological process (BP), cellular component (CC), and molecular function (MF). Pathway analysis was performed using the KEGG database (http://www.genome.jp/kegg/). Secretory protein prediction and functional annotation were performed using SignalP 4.1 (http://www.cbs.dtu.dk/services/SignalP/), SecretomeP 2.0 (http://www.cbs.dtu.dk/services/SecretomeP/), and TMHMM, server 2.0 (http://www.cbs.dtu.dk/services/TMHMM/). Ingenuity pathway analysis (IPA) was used to conduct functional analysis (Ingenuity Systems, http://www.ingenuity.com/). The plasma proteome database (PPD) was used to confirm the association between the proteins that were identified in human plasma and the proteins that were identified in this study [54, 55]. The proteins that were identified in our dataset were crossreferenced with mRNA and protein expression in pancreatic sections in the Human Protein Atlas (http://www.proteinatlas.org/).

### Western blot analysis

A total of 19 pancreatic cyst fluid samples—10 LGD, 4 HGD, and five invasive IPMN—were used to validate the candidate markers. Equal volumes of a pooled cyst fluid sample were loaded onto each gel to correct for the intensity of the blots. Pancreatic cyst fluid samples were mixed with 5× SDS loading dye (250 mM Tris–Cl, pH 6.8, 10% SDS, 50% glycerol, 0.5 M DTT, 0.1% bromophenol blue). Proteins (40 μg, as measured by BCA assay) were separated on 10% SDS-PAGE gels and transferred to polyvinylidene fluoride (PVDF) membranes (Hybond-P, GE Healthcare, Pittsburgh, PA, USA). The membranes were stained with Ponceau S dye (P7170, Sigma-Aldrich, USA), blocked with 5% BSA for 2 h at RT, and incubated overnight at 4 °C with the following primary antibodies: rabbit monoclonal anti-HOOK1 (ab150397, Abcam, Cambridge, UK) at 1:250, mouse monoclonal anti-PTPN6 (sc-7289, Santa Cruz Biotechnology, USA) at 1:1000, and mouse polyclonal anti-SERPINA5 (ab67368, Abcam, Cambridge, UK) at 1:100. The membranes were then washed five times with Tris-buffered saline and Tween-20 (TBS-T) before being incubated with the following HRP-conjugated secondary antibodies: anti-rabbit (ab6721, Abcam, Cambridge, UK) at 1:1000 and anti-mouse (ab6789, Abcam, Cambridge, UK) at 1:2500 for 2 h at RT. The membranes were developed with ECL solution (West-Q chemiluminescent substrate Kit-plus, GenDEPOT, Barker, TX, USA), and the signals were visualized on an LAS-4000 (Fujifilm, Japan).

## Results

### Clinical sample characterization

Pancreatic cyst fluid samples from nine patients were classified into three groups: LGD (n = 3), HGD (n = 3), and invasive IPMN (n = 3). The samples did not differ significantly in composition, with the exception of serum CEA level and CA 19-9 concentration measured by chemiluminescent microparticle immunoassay and cyst size (Table 1). The invasive IPMN patient group had the highest average CEA and CA19-9 concentrations at 7.67 ± 7.06 and 117.17 ± 142.78 mg/L, respectively. CEA and CA19-9 levels were generally higher in the more severe forms of IPMN. The average CEA concentration was approximately 3-fold higher for HGD than LGD subjects and 7-fold higher in invasive IPMN versus LGD. In addition, the average CA19-9 level was approximately 2-fold and 30-fold greater for these comparisons. Our samples were consistent with several publications that have reported that malignant cysts tend to be larger, as evidenced by our invasive IPMN samples (6.63 ± 3.74 cm) being twice as large as LGD (2.93 ± 0.54 cm) and HGD (2.50 ± 0.41 cm) samples on average [5, 56–59].

**Table 1:** Demographic and clinical characteristics of the study population used in the label-free quantification

Group	Pancreatic cyst fluids		
	LGD (*n *= 3)	HGD (*n *= 3)	Invasive IPMN (*n *= 3)
Age (years)	69.00 ± 1.41	66.33 ± 8.58	58.33 ± 11.09
Gender			
Male	1	2	1
Female	2	1	2
Gland type			
Gastric	2	2	1
Intestinal	0	1	1
Oncocytic	0	0	1
Unknown	1	0	0
Duct type			
Main	0	0	1
Branch	2	1	0
Mixed	0	2	2
Unknown	1	0	0
Cyst focality			
Single	2	3	3
Multiple	1	0	0
Unknown	0	0	0
Mural nodule			
Y	0	3	3
N	3	0	0
Unknown	0	0	0
Cyst location			
Head	1	0	2
Body/tail	1	3	1
Mixed	1	0	0
CEA concentration (mg/L)	1.13 ± 0.53	3.07 ± 1.27	7.67 ± 7.06
CA 19-9 concentration (mg/L)	4.00 ± 1.48	6.87 ± 7.17	117.17 ± 142.78
Cyst size			
Cyst size (cm)	2.93 ± 0.54	2.50 ± 0.41	6.63 ± 3.74
< 3.0	1	2	1
≥ 3.0	2	1	2

### In-depth analysis of pancreatic cyst fluid

**Fig. 1:**
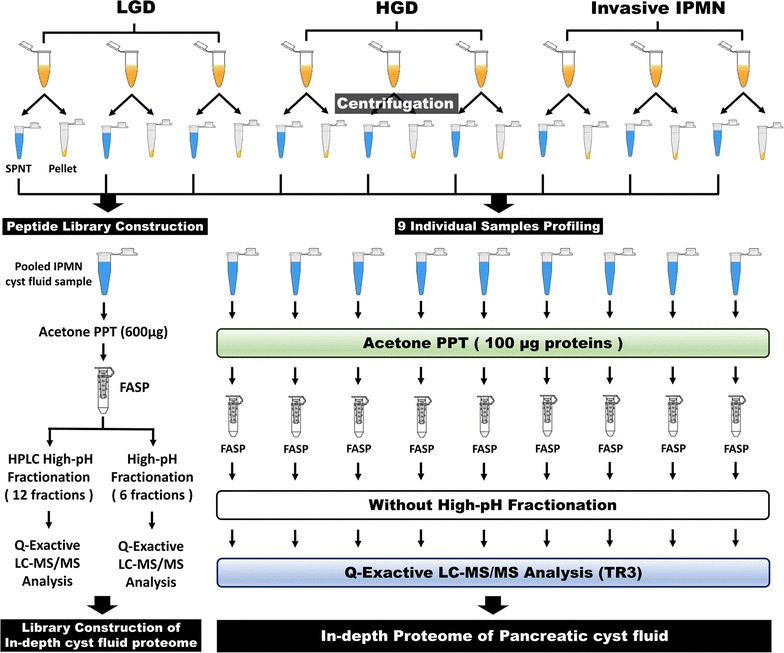
Detailed experimental workflow. Pancreatic cyst fluid samples from nine individuals (3 LGD, 3 HGD, and 3 invasive IPMN) were included in this study. After centrifugation, pellets and debris were discarded, and the supernatant was collected for proteomic analysis. Proteins in each individual sample and pooled cyst fluid (comprised of equal portions of individual samples) were precipitated with cold acetone. Following FASP digestion, the pooled cyst fluids were fractionated using two types of high-pH fractionation methods. Every prepared sample was analyzed on a Q Exactive mass spectrometer

The overall scheme of the study was based on a proteomic platform of cyst fluids that we established earlier [38]. In this study, nine individual pancreatic cyst fluid samples of various types [LGD (n = 3), HGD (n = 3), and invasive IPMN (n = 3)] were used for label-free quantification. All samples were centrifuged, and only the supernatant was used. The same portions of individual samples were pooled and fractionated to generate a peptide library, which increased the depth of the identified proteins. In contrast, the nine individual samples were not fractionated. After a series of sample preparation steps, LC–MS/MS analysis was performed on a Q Exactive mass spectrometer. The MS data were processed in MaxQuant (version 1.5.5.1), and the statistical analysis was performed in Perseus (version 1.5.8.5) (Fig. 1; Additional file 1: Fig. S1a).

**Fig. 2:**
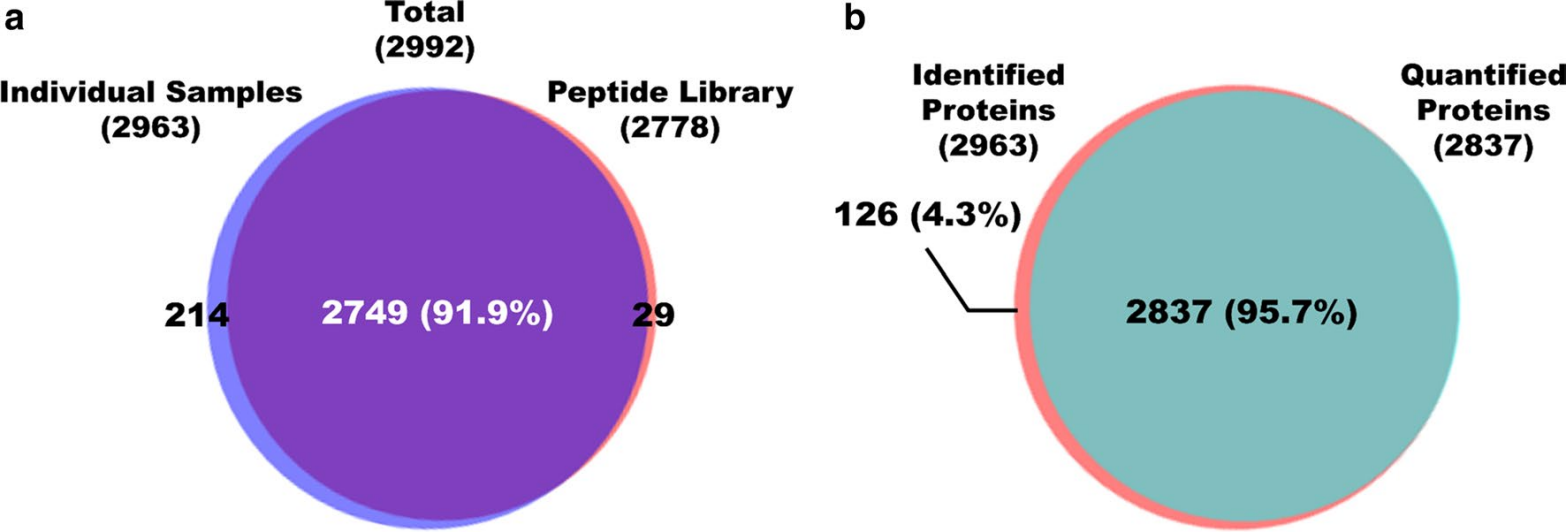
Overlap of identified proteins in the individual samples and peptide library and comparison of identified and quantified proteins. **a** All identified proteins in the nine individual samples and peptide library; 91.9% of proteins were identified both in the individual samples and peptide library. **b** All identified proteins and quantified proteins in the nine individual samples; 95.7% of quantifiable proteins overlapped with the identified proteins

In total, 2992 proteins were identified and 2938 proteins were quantified (Additional file 2: Table S1). A total of 28,728 peptides were identified, and 553,199 peptide spectra matches were found. In the peptide library and the nine individual samples, 2778 and 2963 proteins were identified, respectively. Comparing the peptide library with the individual samples, 2749 proteins (91.9% of all identified proteins) were shared (Fig. 2a). In the nine individual samples, most of the identified proteins (95.7%) were usable for quantitative analysis, as evidenced by the 2963 and 2837 proteins that were identified and quantified (Fig. 2b). Approximately 2200–2500 proteins were quantified in each sample group. The three IPMN groups were similar with regard to the number of quantified proteins (Additional file 1: Fig. S1b). In contrast, there was substantial individual variation in the number of identified and quantified proteins within the same histological subgroups. This pattern was observed across all nine samples (Additional file 1: Fig. S1c).

**Fig. 3:**
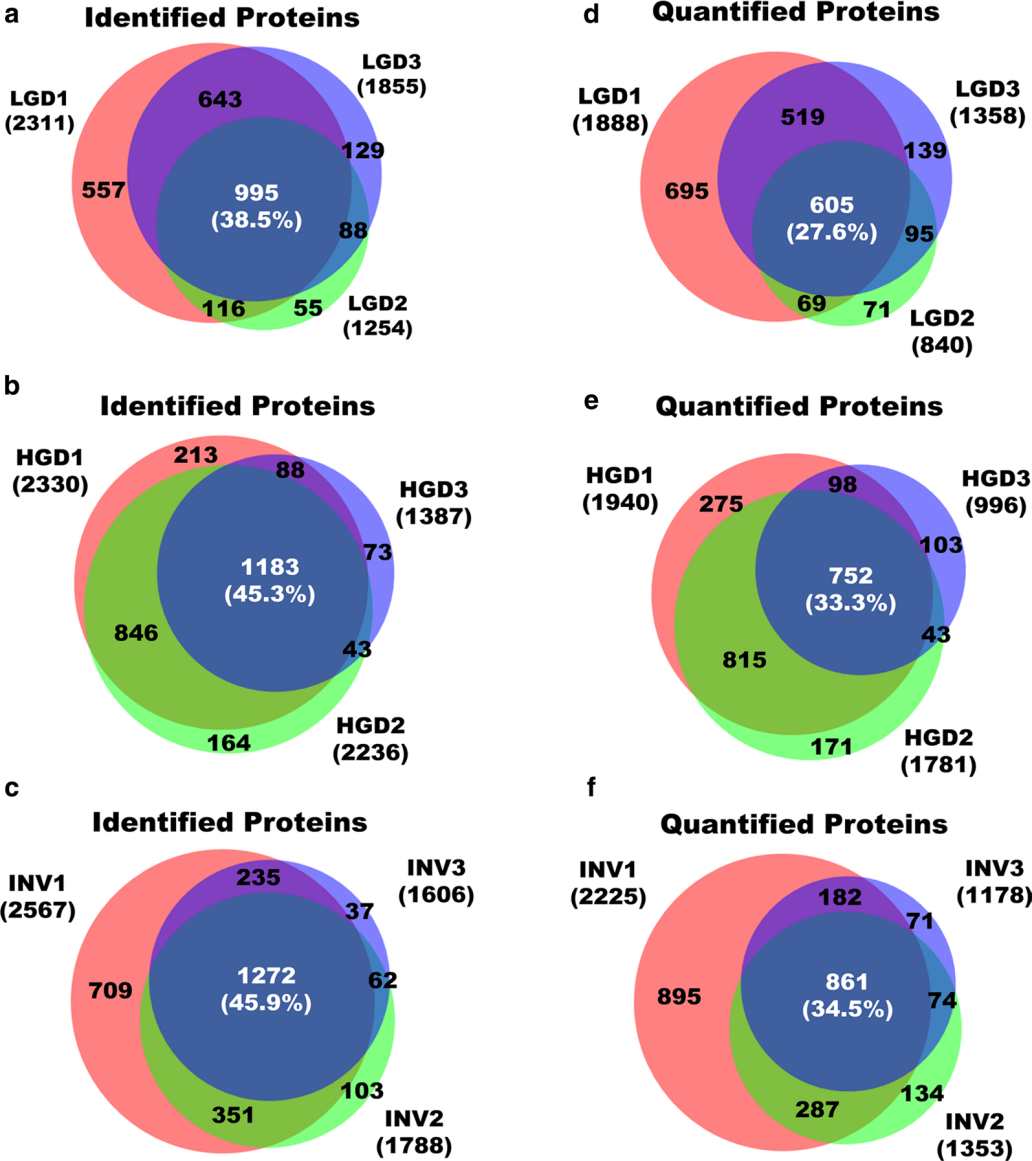
Identified and quantified proteins in three individual samples for each histological grade. Identified proteins of three biological replicates in LGD (**a**), HGD (**b**), and invasive IPMN (**c**). Quantified proteins of three biological replicates in LGD (**d**), HGD (**e**), and invasive IPMN (**f**) (INV: invasive IPMN)

**Fig. 4:**
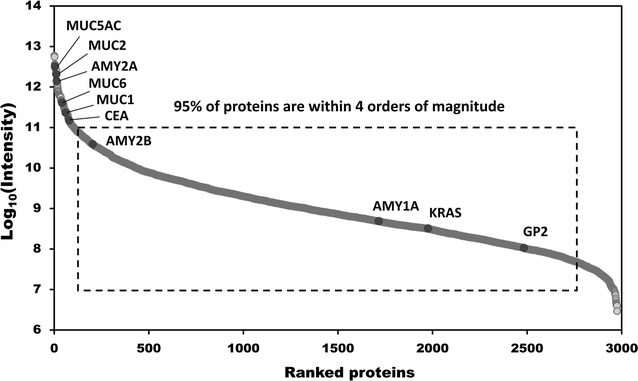
Dynamic range of quantified proteins. Distribution of expression intensities of quantified proteins show a large dynamic range of abundance, but 95% of the proteins were expressed within four orders of magnitude. Several tumor marker proteins, such as MUC2, CEA, and KRAS, were quantified

On average, the number of identified and quantified proteins increased by 129 and 83, respectively, in individual samples when matched with the peptide library. In addition, the number of identified peptides rose by 752 on average in individual samples with HGD 1 displaying the greatest improvement of 2109. (Additional file 3: Table S2). As shown by the Venn diagram, approximately 77% of identified and 63% of quantified proteins overlapped in all histological groups and 337 additional proteins were identified exclusively when the search was performed with the generated peptide library. Whereas the number of proteins that overlapped in the three histological groups decreased by approximately 6% when searched without the peptide library (Additional file 1: Fig. S1d, e, Additional file 2: Table S1). This result implies that the number of proteins that were common between individual samples rose due to the contribution of the peptide library. As shown in Fig. 3, the percentage of overlapping proteins from the biological replicates in each histological grade ranged from 27 to 46%. The dynamic range of the proteome spanned over seven orders of magnitude overall, but most proteins (95%) were expressed within four orders (Fig. 4). Overall, the proteins with lower orders of magnitude were analyzed, and tumor marker proteins, such as MUC5AC, MUC1, and CEA, were quantified with high intensity in the dataset.

### Comparative analysis using other proteome databases

Our bioinformatics analysis showed that secreted proteins accounted for 60.5% (1810 proteins) of the 2992 identified proteins (Additional file 1: Fig. S2a, Additional file 2: Table S1). Across SecretomeP, SignalP, and TMHMM, 1527, 682, and 381 proteins were identified, respectively (Additional file 1: Fig. S2b, Additional file 2: Table S1). Protein accession numbers were converted into gene names, and the resulting redundancy was discarded prior to comparative analysis. We compared our dataset with the Human Plasma Proteome Database to assess the likelihood that the discovered proteins are potential blood markers [54, 55]. As a result, 2299 (79.7%) of the identified proteins were plasma proteins (Additional file 1: Fig. S2c, Additional file 2: Table S1). To determine whether the discovered proteins are expressed in the pancreas, the dataset was crossreferenced with The Human Protein Atlas (http://www.proteinatlas.org, May 31, 2017)—2613 genes had corresponding mRNA entries and 2021 genes had corresponding protein entries in the pancreas (Additional file 1: Fig. S2e, Additional file 2: Table S1).

### Variation in individual cyst samples

**Fig. 5:**
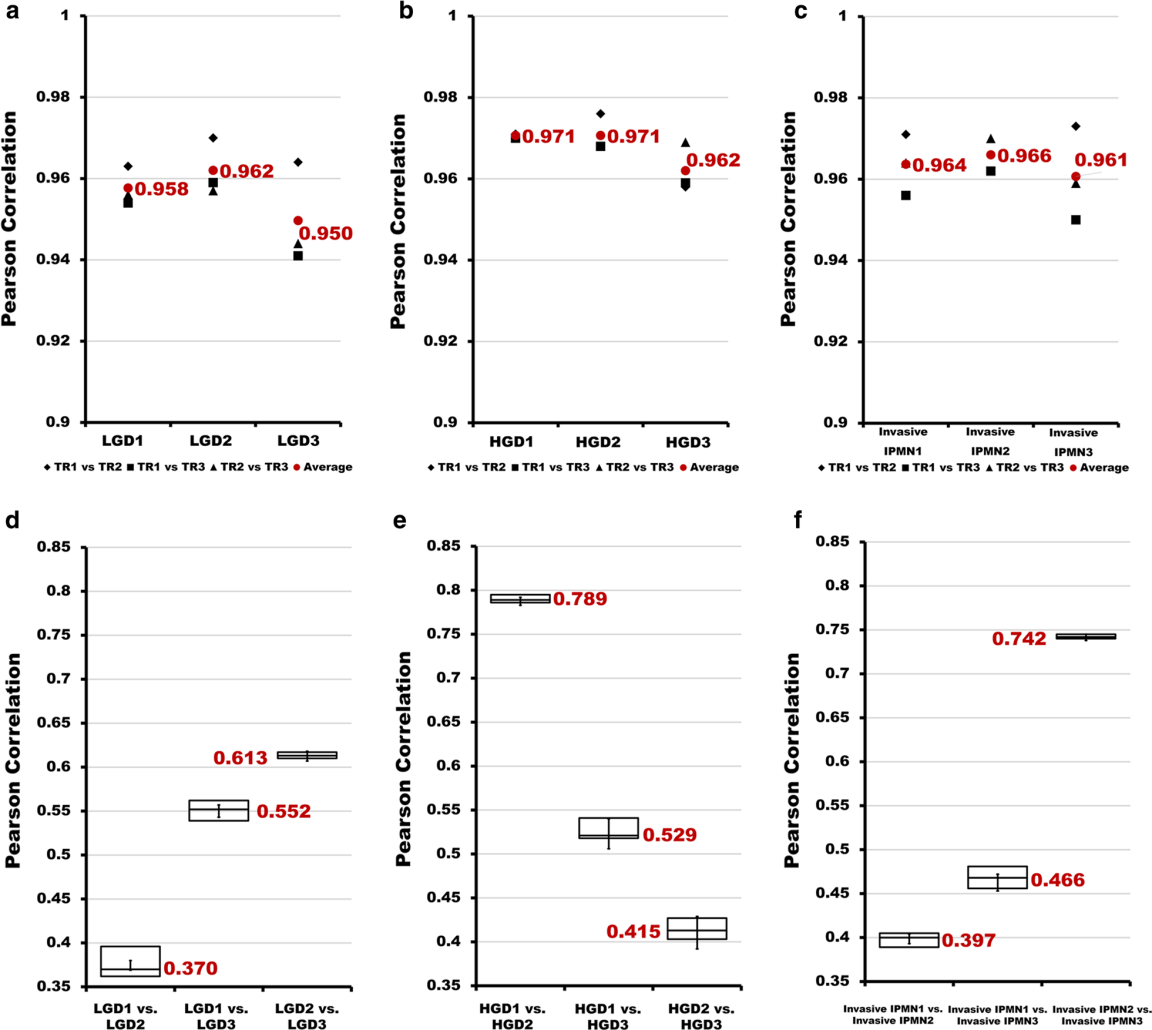
Pearson correlation coefficients of technical replicates (TRs) and biological replicates in each histological group. **a**–**c** Pearson correlation coefficients of technical replicates in each histological group. Three types of marks indicate the Pearson correlation coefficients of each comparison between technical replicates (◆TR1 vs. TR2, ■TR1 vs. TR3, ▲TR2 vs. TR3). Red dots represent the average Pearson correlation coefficient in each of the 3 comparisons. **a**–**c** indicate LGD, HGD, and invasive IPMN, respectively. Box plot representation of Pearson correlation coefficients between each biological replicate in LGD (**d**), HGD (**e**), and invasive IPMN (**f**)

Coefficient of variation (CV%) values were calculated to evaluate the reproducibility of the technical and biological replicates. The CV% values of log2-transformed LFQ intensity sums of technical triplicates of individual samples ranged from 0.32 to 6.45% (Additional file 4: Table S3). All CV% values of log2-transformed LFQ intensities of each quantified protein in technical triplicates of individual samples were less than 20%. Moreover, the average CV% value of individual samples ranged from 1.085 to 1.524% (Additional file 1: Fig. S3a). Pearson correlation coefficients of the LFQ intensities of technical triplicates and their averages were greater than 0.9 (Fig. 5a–c, Additional file 1: Fig. S3b–d). These data suggest that the variation between technical replicates was low. In contrast, the variation between biological triplicates was generally high, based on the Pearson correlation coefficients, which ranged from 0.370 (between LGD1 and LGD2) to 0.789 (between HGD1 and HGD2) (Fig. 5d–f, Additional file 1: Fig. S3b–d).

### Differentially expressed proteins in IPMN dysplasia

**Fig. 6:**
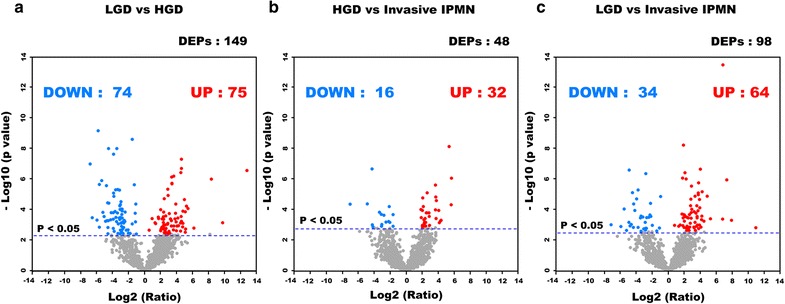
Volcano plots based on *p* values in all comparison groups. To determine the differentially expressed proteins, Student’s *t* test was performed with a Benjamini–Hochberg FDR value of 0.05. The colored dots indicate the proteins that passed the *t* test for significance between LGD versus HGD (**a**), HGD versus invasive IPMN (**b**), and LGD versus invasive IPMN (**c**). The blue dots represent downregulated proteins, and the red dots denote upregulated proteins

The 1751 proteins that had at least six valid values within a histological group were used for the statistical analysis (Additional file 5: Table S4). By Student’s *t* test (Benjamini–Hochberg FDR = 0.05), 149, 48, and 98 proteins were differentially expressed between comparisons 1 (LGD versus HGD), 2 (HGD versus invasive IPMN), and 3 (LGD versus invasive IPMN), respectively (Additional file 5: Table S4, Fig. 6), 75, 32, and 64 of which were upregulated. By unsupervised hierarchical clustering, the DEPs clustered by IPMN histology (Additional file 1: Fig. S4).

**Fig. 7:**
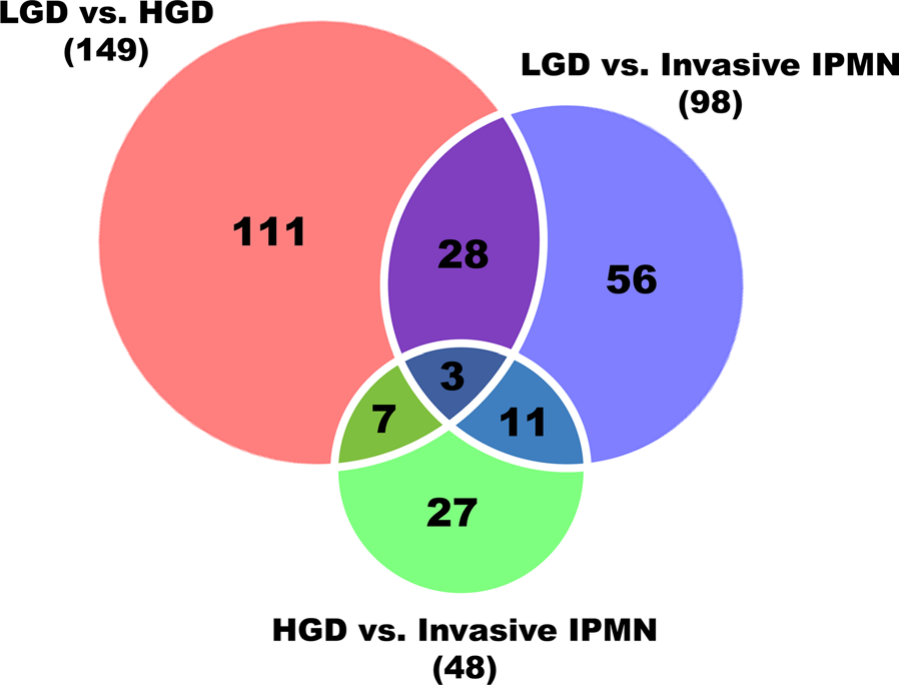
Venn diagram of differentially expressed proteins in three comparison groups. By Student’s *t* test (Benjamini–Hochberg FDR = 0.05), 149, 48, and 98 proteins were differentially expressed between LGD and HGD, between HGD and invasive IPMN, and between LGD and invasive IPMN, respectively. A total 243 proteins were found to be DEPs when overlapping components of the Venn diagram were excluded; 49 proteins were shared in at least two comparison groups and are highlighted in white

There were 243 DEPs across comparisons 1, 2, and 3. Among the 243 DEPs (Fig. 7), 142 were upregulated and 91 were downregulated in at least 1 comparison group (Additional file 6: Table S5). Enriched DEPs were used to conduct GO and KEGG pathway analyses to identify their overrepresented terms in biological process, molecular function, and cellular component. The DEPs from comparisons 1 and 3 were analyzed by ingenuity pathway analysis (IPA) bioinformatics tool to track biological processes that became activated or more pronounced in aggressive malignancy.

By GO enrichment analysis, 243 DEPs were involved primarily in vesicle-mediated transport and cell surface receptor signaling with regard to biological process. The analysis also found that 76.6% of DEPs were extracellular region proteins. The molecular functions of the DEPs were primarily associated with peptidase activity and regulation (Additional file 1: Fig. S5a–c, Additional file 7: Table S6). By KEGG pathway analysis, the 142 upregulated proteins were associated with the ribosome, oxidative phosphorylation, and endocytosis, whereas the 91 downregulated proteins were linked to leukocyte transendothelial migration, focal adhesion, and ECM-receptor interaction (Additional file 1: Fig. S5d).

The significantly changed proteins from comparison 1 and 3 were examined by IPA with regard to biological processes that are related to pancreatic cysts and aggressive malignancy. Core analysis in IPA was used to evaluate the biological functions that were most likely to be affected by changes in expression of proteins in our dataset. As a result, 149 DEPs in comparison 1 and 98 DEPs in comparison 3 were associated with such terms as cellular movement and angiogenesis in Diseases and Functions, which are indicative of malignancy; the biological terms that correlated with aggressive malignancy are highlighted in yellow (Additional file 1: Fig. S6a, b). Cell growth and vasculogenesis were upregulated among the DEPs in comparison 1. A total of 98 DEPs in comparison 3 were upregulated in most Diseases and Functions terms, except for apoptosis of tumor cell lines—particularly metastasis-related terms, such as cell spreading and angiogenesis.

Comparison analysis is usually performed to visualize trends in protein expression across several analyses. Consistent with our expectations, the Diseases and Bio functions heat map of the comparison analysis demonstrated that the DEPs that were associated with cell movement of endothelial cells and angiogenesis were more highly expressed in comparison 3 versus 1. The term “apoptosis of tumor cell lines” was downregulated in comparison 3 but unchanged in comparison 1 (Additional file 1: Fig. S6c). A higher percentage of DEPs in comparison 3 was associated with pancreas-specific diseases, such as chronic pancreatitis and associated diseases than DEPs in comparison 1 (Additional file 1: Fig. S6d).

### Biomarker candidates for IPMN malignancy

Proteins that were shared by at least two comparison groups were chosen as the initial marker candidates. Our rationale was that significantly changed proteins that are common to several comparison groups are more likely to be associated with the malignancy of IPMNs [60]. A total of 49 candidates expressed in at least two comparison groups were selected from 243 DEPs (Fig. 7). Then, the DEPs that had expression patterns that were consistent with the degree of IPMN malignancy were selected as the final candidates. Table 2 details the results of the statistical analysis of the 49 DEPs, including the *p* value, fold-change, and *t* test significance for each comparison group. Of the 49 DEPs, 38 were secreted proteins and 33 were confirmed to be expressed in the pancreas as mRNA or proteins in The Human Protein Atlas. In addition, 35 proteins were confirmed to be expressed in plasma, according to the Human Plasma Proteome Database (Table 2).

**Table 2:** Detailed statistical analysis of 49 DEPs that were shared in at least two comparison groups

Protein name	LGD versus HGD			HGD versus invasive IPMN			LGD versus invasive IPMN		
	*t* test significance	*p* value	Fold-change	*t* test significance	*p* value	Fold-change	*t* test significance	*p* value	Fold-change
EH domain-containing protein 1	+	0.00594	− 15.43	+	0.01955	7.35		0.54376	− 2.10
Immunoglobulin lambda variable 3–21	+	0.00586	− 18.35	+	0.00001	41.24		0.54057	2.25
Collagen alpha-1(VI) chain	+	0.00224	− 22.78	+	0.01403	5.61		0.19350	− 4.06
Vacuolar protein sorting-associated protein 37B	+	0.01350	− 6.78	+	0.04669	4.56		0.76562	− 1.49
Ig lambda-6 chain C region	+	0.03265	− 4.96	+	0.02993	20.47		0.31759	4.13
Ras-related protein Ral-B	+	0.00571	− 5.53	+	0.00111	12.71		0.33130	2.30
Lysozyme C	+	0.03497	3.95	+	0.03528	− 5.79		0.66323	− 1.47
Immunoglobulin heavy variable 1/OR15-1		0.00000	1.00	+	0.02490	5.06	+	0.00046	7.47
Immunoglobulin lambda variable 5–45		0.93977	− 1.15	+	0.03513	17.43	+	0.02740	15.11
^b^Aldo–keto reductase family 1 member B10		0.97902	− 1.08	+	0.00808	− 131.01	+	0.02709	− 140.91
Ig heavy chain V–III region TIL		0.22132	− 1.77	+	0.00288	6.15	+	0.00022	3.48
Ig heavy chain V–III region BUT		0.61619	1.58	+	0.01276	4.09	+	0.03193	6.47
Ig heavy chain V–III region BUR		0.61583	1.72	+	0.03530	5.43	+	0.03177	9.36
Ig heavy chain V–III region ZAP		0.81620	1.29	+	0.01443	6.03	+	0.00587	7.80
Ig alpha-2 chain C region		0.14392	− 2.13	+	0.02303	3.70	+	0.02778	1.74
^a^Thymidine phosphorylase		0.89045	− 1.25	+	0.00774	48.91	+	0.01333	39.00
^a^Testis-expressed sequence 12 protein		0.75598	− 1.40	+	0.01204	16.69	+	0.00379	11.94
^a^Protein Hook homolog 1		0.33603	2.29	+	0.00053	50.05	+	0.00000	114.50
Collagen alpha-1(XV) chain	+	0.00001	− 11.37		0.54289	1.75	+	0.01252	− 6.49
Prosaposin	+	0.01353	− 2.27		0.69798	1.16	+	0.00131	− 1.95
^b^Phosphoinositide-3-kinase-interacting protein 1	+	0.00707	− 7.41		0.00000	1.00	+	0.01999	− 7.35
^b^Thy-1 membrane glycoprotein	+	0.00966	− 8.39		0.00000	1.00	+	0.01176	− 10.48
Latent-transforming growth factor beta-binding protein 2	+	0.01295	− 30.47		0.83621	1.13	+	0.02886	− 26.88
Protein disulfide-isomerase A3	+	0.00534	27.04		0.64689	− 1.78	+	0.00176	15.23
Agrin	+	0.01010	− 95.04		0.64723	1.58	+	0.03007	− 59.97
Alpha-1-antichymotrypsin	+	0.00041	− 8.93		0.52129	1.65	+	0.01226	− 5.40
Collagen alpha-1(IV) chain	+	0.00000	− 23.08		0.36550	2.54	+	0.01235	− 9.09
^b^Trefoil factor 1	+	0.03148	− 4.27		0.32370	− 4.75	+	0.03214	− 20.28
^b^Plasma serine protease inhibitor	+	0.00000	− 3.00		0.07044	− 2.33	+	0.00013	− 7.00
Guanine nucleotide-binding protein G(k) subunit alpha	+	0.00446	− 7.09		0.57151	1.44	+	0.01032	− 4.92
Guanine nucleotide-binding protein G(k) subunit alpha	+	0.00042	− 12.58		0.94988	− 1.07	+	0.00077	− 13.45
Collagen alpha-3(VI) chain	+	0.00016	− 41.81		0.61089	2.11	+	0.00316	− 19.85
^a^Tyrosine-protein phosphatase non-receptor type 6	+	0.02363	4.39		0.50095	1.58	+	0.00391	6.95
^b^Kallistatin	+	0.01495	− 6.30		0.68656	1.42	+	0.01178	− 4.45
^b^Fibrillin-1	+	0.00000	− 57.02		0.43700	1.93	+	0.00012	− 29.49
^b^Claudin-18	+	0.00001	− 14.94		0.67515	− 1.49	+	0.00101	− 22.20
Coronin-7	+	0.00858	6.09		0.85877	− 1.18	+	0.01207	5.15
^a^Nuclease-sensitive element-binding protein 1	+	0.04238	6.17		0.62145	1.67	+	0.00138	10.30
Histone H3.1	+	0.00023	10.62		0.25248	− 2.30	+	0.01205	4.62
^b^Mucin-5AC	+	0.02741	− 15.38		0.69722	− 1.94	+	0.02010	− 29.87
^a^Mucin-2	+	0.00006	7068.07		0.76813	− 3.54	+	0.03333	1996.84
^b^WAP four-disulfide core domain protein 2	+	0.00554	− 13.23		0.83040	− 1.38	+	0.00690	− 18.23
^b^Cystatin-M	+	0.01152	− 19.49		0.00000	1.00	+	0.03235	− 13.72
BPI fold-containing family B member 1	+	0.00026	− 27.59		0.49944	1.78	+	0.00180	− 15.53
Intelectin-1	+	0.01505	844.39		0.76240	− 3.48	+	0.01605	242.93
^a^Talin-1	+	0.03653	8.08		0.60098	1.46	+	0.02980	11.78
Basement membrane-specific heparan sulfate proteoglycan core protein	+	0.00003	− 114.37	+	0.00582	13.69	+	0.03552	− 8.35
Histone H3	+	0.00007	22.86	+	0.01650	− 3.15	+	0.00843	7.26
Cadherin-related family member 2	+	0.00024	− 52.14	+	0.02477	13.15	+	0.02747	− 3.96

Protein name	SignalP	SecretomeP	TMHMM	The Human Protein Atlas		Human plasma proteome
	Passed	Passed	Passed	RNA expression	Protein expression	Included
EH domain-containing protein 1	–	–	–	Y	–	Y
Immunoglobulin lambda variable 3–21	Y	Y	–	–	–	–
Collagen alpha-1(VI) chain	Y	–	–	Y	–	Y
Vacuolar protein sorting-associated protein 37B	–	–	–	Y	Y	Y
Ig lambda-6 chain C region	–	Y	–	–	–	–
Ras-related protein Ral-B	–	–	–	Y	Y	–
Lysozyme C	Y	Y	–	Y	Y	Y
Immunoglobulin heavy variable 1/OR15-1	Y	Y	–	–	–	–
Immunoglobulin lambda variable 5–45	Y	Y	–	–	–	–
^b^Aldo–keto reductase family 1 member B10	–	–	–	–	–	–
Ig heavy chain V–III region TIL	–	Y	–	–	–	–
Ig heavy chain V–III region BUT	–	Y	–	–	–	–
Ig heavy chain V–III region BUR	–	Y	–	–	–	–
Ig heavy chain V–III region ZAP	–	–	–	–	–	–
Ig alpha-2 chain C region	–	Y	–	–	–	Y
^a^Thymidine phosphorylase	–	Y	–	Y	–	Y
^a^Testis-expressed sequence 12 protein	–	Y	–	–	–	Y
^a^Protein Hook homolog 1	–	–	–	Y	–	Y
Collagen alpha-1(XV) chain	–	–	–	Y	–	Y
Prosaposin	Y	Y	–	Y	Y	Y
^b^Phosphoinositide-3-kinase-interacting protein 1	Y	–	–	Y	–	Y
^b^Thy-1 membrane glycoprotein	Y	–	–	Y	–	Y
Latent-transforming growth factor beta-binding protein 2	Y	–	–	Y	–	Y
Protein disulfide-isomerase A3	–	–	–	Y	Y	Y
Agrin	Y	–	–	Y	–	Y
Alpha-1-antichymotrypsin	Y	Y	–	Y	Y	Y
Collagen alpha-1(IV) chain	Y	–	–	Y	–	Y
^b^Trefoil factor 1	Y	Y	–	Y	–	Y
^b^Plasma serine protease inhibitor	Y	Y	–	Y	–	Y
Guanine nucleotide-binding protein G(k) subunit alpha	–	Y	–	Y	Y	Y
Guanine nucleotide-binding protein G(k) subunit alpha	Y	–	–	Y	Y	Y
Collagen alpha-3(VI) chain	Y	–	–	Y	–	Y
^a^Tyrosine-protein phosphatase non-receptor type 6	–	–	–	Y	–	Y
^b^Kallistatin	Y	Y	–	Y	Y	Y
^b^Fibrillin-1	Y	–	–	Y	–	Y
^b^Claudin-18	–	–	Y	Y	–	–
Coronin-7	–	–	–	Y	Y	Y
^a^Nuclease-sensitive element-binding protein 1	–	Y	–	Y	Y	Y
Histone H3.1	–	Y	–	–	Y	–
^b^Mucin-5AC	Y	–	–	–	–	–
^a^Mucin-2	Y	–	–	–	–	Y
^b^WAP four-disulfide core domain protein 2	Y	Y	Y	Y	–	Y
^b^Cystatin-M	Y	Y	–	–	–	Y
BPI fold-containing family B member 1	Y	Y	Y	–	–	Y
Intelectin-1	Y	Y	–	Y	–	Y
^a^Talin-1	–	–	–	Y	Y	Y
Basement membrane-specific heparan sulfate proteoglycan core protein	Y	–	–	Y	Y	Y
Histone H3	–	Y	–	–	Y	–
Cadherin-related family member 2	Y	Y	Y	–	–	Y

^a^Dominantly expressed in invasive IPMN

^b^Dominantly expressed in LGD

**Fig. 8:**
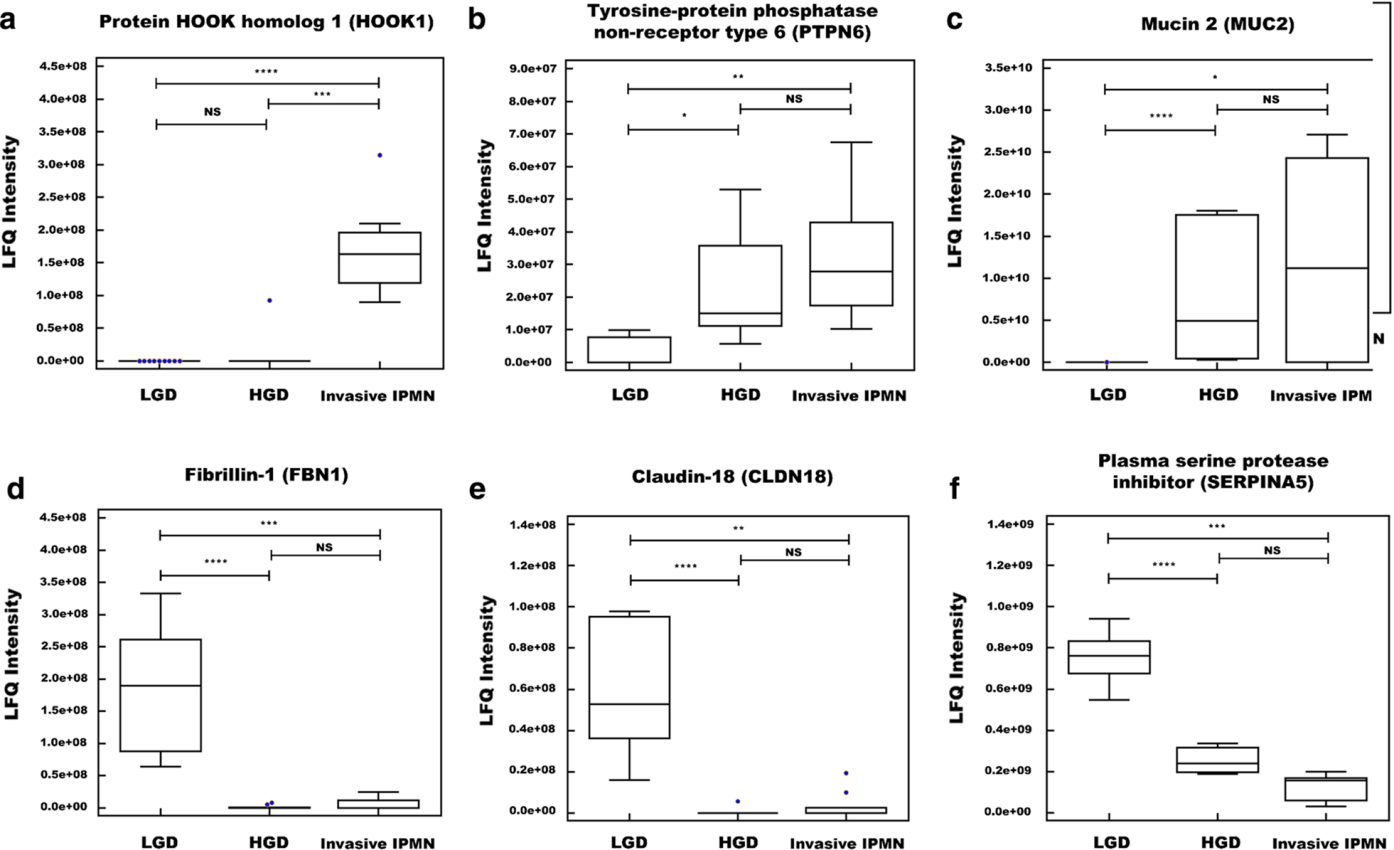
Six biomarker candidates among 18 proteins that had expression patterns that were consistent with the degree of IPMN malignancy. HOOK1 (**a**), PTPN6 (**b**), and MUC2 (**c**) were predominantly expressed in invasive IPMN. FBN1 (**d**), CLDN18 (**e**), and SERPINA5 (**f**) were primarily expressed in LGD (*<*p* value 0.05; **<*p* value 0.01, ***<*p* value 0.001, ****<*p* value 0.0001, *NS* not significant)

Of the 49 shared DEPs between groups, 18 had expression patterns that were consistent with the degree of malignancy. PTPN6, MUC2, TLN1, and YBX1 were expressed in lower amounts in LGD but gradually elevated in HGD and invasive IPMN. Conversely, SERPINA5, AKR1B10, and TFF1 expression decreased as IPMN histological grade progressed. Other proteins, such as HOOK1, TYMP, TEX12, FBN1, CLDN18, THY1, MUC5AC, CST6, WFDC2, PIK3IP1, and SERPINA4, were predominantly expressed in LGD or invasive IPMN but not in other groups (Fig. 8, Additional file 1: Fig. S7). Based on these results, these 18 proteins were selected as potential biomarkers of IPMN dysplasia.

### Validation by western blot

**Fig. 9:**
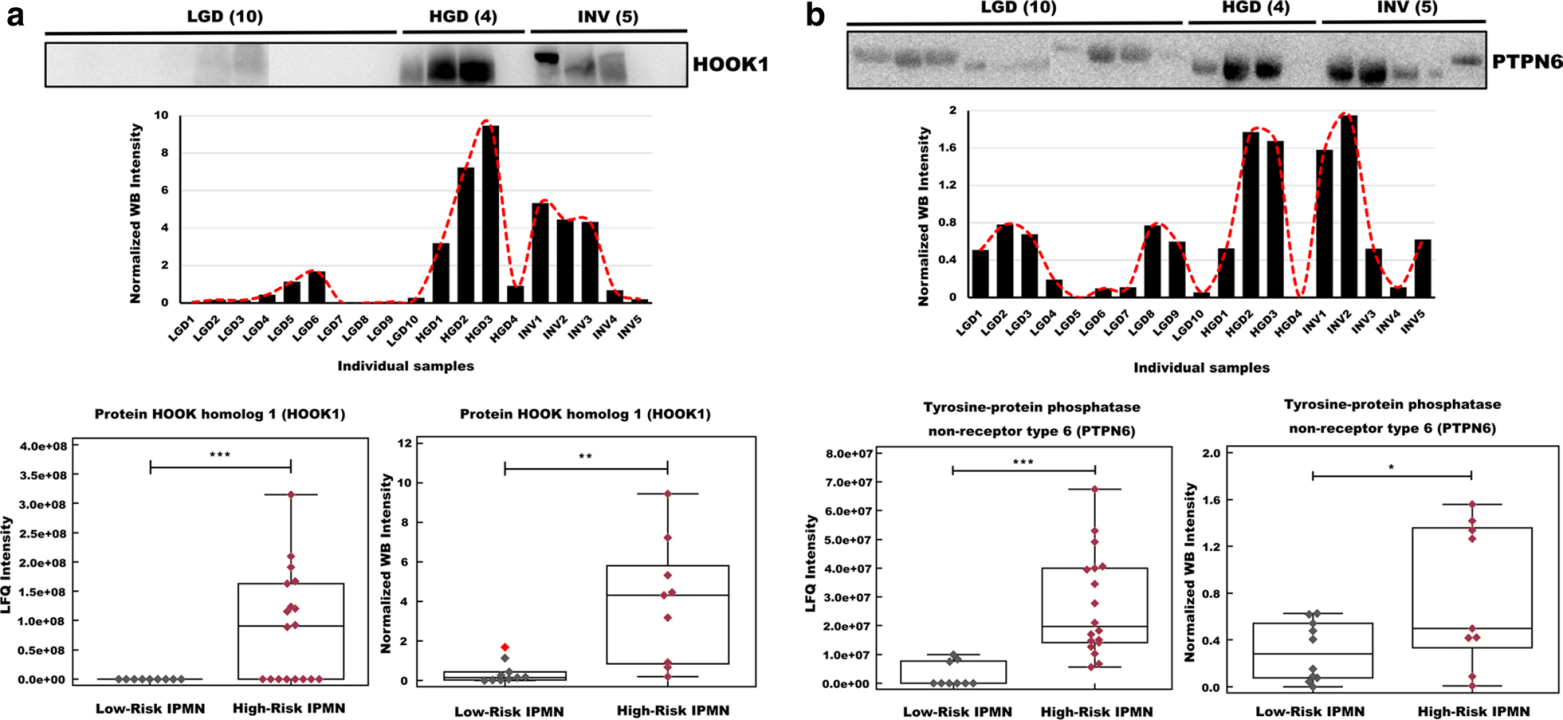
Validation of HOOK1 and PTPN6 as potential biomarker targets by western blot. A total of 19 pancreatic cyst fluid samples were analyzed by western blot to validate the relative abundance of HOOK1 and PTPN6. The immunoblotting results were consistent with our MS results. **a** HOOK1 was overexpressed in high-risk IPMN in the proteomic (*p* value < 0.001) and western blot analyses (*p* value < 0.01). **b** PTPN6 was overexpressed in high-risk IPMN in the proteomic (*p* value < 0.001) and western blot analyses (*p* value < 0.05) (*<*p* value 0.05, **<*p* value 0.01, ***<*p* value 0.001, ****<*p* value 0.0001, *NS* not significant, *INV* invasive IPMN)

Two DEPs (HOOK1 and PTPN6) were validated by western blot using 19 pancreatic cyst fluid samples (10 LGD, 4 HGD, and 5 invasive IPMN). Patient information including demographics, cyst characteristics, and CEA and CA19-9 levels are provided in Additional file 8: Table S7. The results were then compared with the MS analysis findings (Fig. 9). Although not every western blot sample followed the trend in the MS analysis, the expression patterns of HOOK1 and PTPN6 generally correlated with the LFQ intensity values. HOOK1 was significantly upregulated in high-risk IPMN (*p* value < 0.01), and PTPN6 was detected at higher levels in high-risk IPMN (*p* value < 0.05).

## Discussion

Most pancreatic neoplasms, which are predominantly IPMN, are discovered incidentally during routine check-ups [10]. Nevertheless, the lack of a standardized guideline adds subjectivity and undesired variability in diagnosing the malignancy of IPMN lesions. Because the concentrations of tumor biomarkers are higher in cyst fluid than in blood, pancreatic cyst fluid of IPMN patients was analyzed to discover biomarker candidates that could address these inconsistencies in diagnosing IPMN malignancy [6, 28]. Thus, analyzing proteins that vary significantly, depending on the malignancy of IPMN, can identify biomarkers that improve the diagnostic performance of current methods and decrease the number of patients who undergo unnecessary operations [12, 20].

As shown in our results, we generated a pancreatic cyst fluid proteome that comprised 2992 proteins (Fig. 2a, Additional file 2: Table S1). Our proteome had three and seven times the number of proteins versus studies by Cuoghi [36] and Gbormittah [37], respectively. Further, 1291 additional proteins were identified over our previous study [38] by optimizing the standard proteomic profiling platform by constructing a peptide library of a pooled sample, methodically preparing samples, and reproducibly performing label-free quantitative analysis in triplicates (Additional file 1: Fig. S2d). Normally, DDA acquisition cannot detect low-abundance proteins in individual samples, because high-abundance proteins saturate the signal. By pooling and fractionating individual samples, these low-abundance proteins became distinct and detectable, as evidenced by a dynamic range that spanned seven orders of magnitude (Fig. 4). Consequently, the number of identified and quantified proteins that were common to all individual samples rose substantially when the mass spectra of individual samples were matched to those of the peptide library (Additional file 1: Fig. S1d, e, Additional file 3: Table S2) [61, 62]. This increase enabled us to select biomarker candidates from a larger pool of DEPs.

Most identified proteins (79.7%) that had entries in the Plasma Proteome Database and all marker candidates in our study, except AKR1B10, CLDN18, and MUC5AC, were confirmed to be expressed in plasma (Additional file 1: Fig. S2c, Table 2). This result suggests that the discovered candidates are potential blood marker candidates. Taking into account that 70.0% of proteins were expressed in the pancreas, according to The Human Protein Atlas, it is probable that the biomarker candidates are specific to the pancreas (Additional file 1: Fig. S2e, Additional file 2: Table S1). Considering the bioinformatics analysis results of secreted proteins, we conclude that secreted proteins that originate from pancreatic epithelial cells constitute a significant portion of cyst fluid (Additional file 1: Fig. S2a, Additional file 2: Table S1). The high percentage of matches in these comparative analyses confirms that virtually all of the debris was discarded and that only cyst fluid was collected during sample preparation.

The high Pearson correlation coefficients (> 0.9) that were obtained from the pairwise correlation analysis of LFQ intensity values indicated a strong correlation between technical triplicates and that the MS data were acquired without bias (Fig. 5a–c, Additional file 1: Fig. S3b–d). In contrast, the Pearson correlation coefficient of the biological replicates of the histology groups was low, as shown in Fig. 5d–f and Additional file 1: Fig. S3b–d, primarily due to the wide variety of cyst types, the variations in cyst size, and the presence of blood contaminants [63–65]. One possible source of variation is the contamination of cyst fluid by blood. Fortunately, the samples in this experiment were relatively clean, as evidenced by the inability to detect albumin and low (intensity rank 1475) IgG levels (Fig. 4, Additional file 2: Table S1). Despite using relatively clean cyst fluid, the variation between individual cyst fluid samples remained large (Additional file 1: Fig. S1c, Fig. 5d–f). Based on this result, we infer that using contaminated samples will result in even greater individual variation.

Selecting proteins that had at least six valid values within a histological group mitigated the likelihood of analyzing proteins that are not representative of their histology group, as evidenced from the low *p* value of the *t* test, the high fold-change value, and the clear division between clusters shown in the heat map (Additional file 1: Fig. S4, Additional file 5: Table S4). After eliminating DEPs that were unique to single comparison group, 18 proteins that changed expression levels in accordance with the degree of IPMN malignancy were selected as biomarker candidates (Table 2, Fig. 8, and Additional file 1: Fig. S7). Overall, our stringent criteria—requiring at least six valid values in a histological group, rigorous statistical analysis parameters, and a consistent expression pattern across histology groups—significantly increased the probability of finding more credible biomarker candidates.

All 18 biomarker candidates were associated with pancreatic disease and malignancy (Additional file 9: Table S8). With the exception of HOOK1, TEX12, TLN1, and PIK3IP1, all candidates are expressed in pancreatic tissue [66–94]. Twelve candidates were associated with pancreatic diseases, such as IPMN, pancreatic ductal adeno carcinoma (PDAC), and pancreatitis [66–79, 81–85, 87, 88, 92–97]. According to Tanaka, CLDN18 is an early-stage marker of PDAC and is elevated in high-grade versus low-grade lesions, consistent with our data [5]. Two types of mucin proteins were selected as biomarker candidates and have been examined in studies on IPMN and pancreatic cancer. Our protein expression patterns were consistent with those of prior studies. One of the two mucin biomarkers, MUC5AC, is expressed at high levels during the early stages of pancreatic ductal dysplasia but is low in high-grade tumors [70, 72]. MUC2 is expressed in IPMNs but not normal pancreatic tissue or PDAC [70].

PTPN6, YBX1, TYMP, CLDN18, WFDC2, SERPINA4, TFF1, MUC2, MUC5AC, CST6, THY1, and AKR1B10 overexpressed in PDAC and pancreatitis. PTPN6 has not been reported in human pancreatic samples but has been observed in PANC-1 cell lines and a rat model of pancreatitis [95, 96]. The upregulated proteins, YBX1 and TYMP, are expressed at higher levels in PDAC versus normal tissue, a pattern that is consistent with our proteomic data [87, 88]. In addition, these candidates are overexpressed in other types of cancer, such as breast and bladder cancer [98–101]. The remaining six candidates, except SERPINA4 and MUC2, are overexpressed in PDAC [66–68, 71, 79, 81, 93]. These proteins are involved in tumor progression and differentiation. Accordingly, they are regarded as marker candidates of various cancer types. WFDC2 is a potential early diagnostic marker of gynecological cancers, such as ovarian and endometrial cancer [102]. Moreover, serum levels of WFDC2 are indicative of ovarian cancer [103]. TFF1, THY1, and AKR1B10 are associated with various cancers and have been implicated as biomarker candidates [104–108]. Although it is unknown whether SERPINA4 mediates the progression of pancreatic cancer, it is an early marker of severity in acute pancreatitis [97]. These studies have demonstrated that our final list comprises bona fide candidate markers for IPMN. Our report has significance as the first study to discover the following marker candidates of IPMN: HOOK1, TEX12, TLN1, SERPINA5, FBN1, and PIK3IP1. With the exception of TEX12, these proteins are associated with other cancers, such as hepatocellular carcinoma, breast cancer, and prostate cancer [109–121]. Considering the literature regarding the 18 candidates, it is likely that they are related to IPMN malignancy, except for TEX12.

In order to confirm the validity of the aforementioned marker candidates, we compared our MS analysis results with western blot results. Western blot with cyst fluids is difficult due to the lack of housekeeping proteins, such as alpha-tubulin and beta-actin. To address this issue, we used 0.1% Ponceau S solution as a loading control (Additional file 1: Fig. S8, Additional file 10: Table S9) [122, 123]. The CV% of the intensities of individual samples was 14.19%, indicating that approximately equal amounts had been loaded onto the SDS-PAGE gels. Three DEPs were selected for further validation: two upregulated (HOOK1 and PTPN6) and one downregulated protein (SERPINA5). The selection criteria for validation were a low *p* value, high LFQ intensities, and a lack of an association with IPMN in the literature (which suggests novelty).

HOOK1 was highly expressed in HGD and invasive IPMN compared with LGD (*p* < 0.01). Although the difference in PTPN6 was not statistically significant between the three comparison groups, its overall expression pattern underwent similar changes as in the MS results (Additional file 1: Fig. S9a, b). The expression pattern of SERPINA5 was not consistent with the MS analysis and was higher in high-risk IPMN (Additional file 1: Fig. S9c). This inconsistency might have resulted from the inherent property of western blots, which depends on the affinity between an antibody and a single antigenic epitope [124–132]. Thus, if the antibody has weak affinity for the epitope, the western blot results would not be an accurate measure of protein abundance. In this regard, although western blot has been the standard assay in proteomics, targeted proteomic analysis might be a better alternative for verifying our quantitative MS data.

## Conclusions

We have identified 2992 proteins in IPMN cyst fluid samples using mass spectrometry techniques. Our investigation demonstrates that the use of a peptide library is beneficial, because the increased number of identified proteins provides a wider selection to choose from as biomarkers. This is evident from our dataset, which contains the largest number of proteins for pancreatic cyst fluid. Our in-depth data on the pancreatic cyst fluid proteome will be a valuable resource for pancreatic disease research.

Our bioinformatics analysis concluded that upregulated DEPs were associated with pancreatic cell proliferation and aggressive malignancy. Through statistical analysis, we designated 18 biomarker candidates that changed expression levels, depending on the histological grade of IPMN. Among them, two upregulated DEPs were consistent with our western blot analysis. The literature has also concluded that these proteins are involved primarily in pancreatic diseases and malignancy, rendering them promising biomarker candidates of IPMN malignancy. In future studies, we plan to collect a sufficient amount of cyst fluid samples from more patients to test the performance of these biomarkers by immunoassay and multiple reaction monitoring (MRM).

## Additional files

**Additional file 1.** Supplementary Figures S1–S9.

**Additional file 2.** Table S1. List of identified proteins. MS information on identified and quantitated proteins is listed consecutively. SignalP and SecretomeP were used to identify the secreted proteins, whereas TMHMM was used to identify transmembrane proteins. The Human Plasma Proteome Database confirmed the association between the proteins that were identified in human plasma and the identified proteins of this study. Identified proteins in our dataset were crossreferenced with pancreatic expression in the Human Protein Atlas. LFQ intensities of individual samples were used for further statistical analysis. The additional proteins searched exclusively in the presence of peptide library were highlighted as the additional column in this table.

**Additional file 3.** Table S2. The number of identified and quantified proteins in individual samples searched with and without the peptide library. The number of identified and quantified proteins increased by 129 and 83, respectively, on average per individual samples when searched with and without the peptide library. In the same manner, the number of identified peptides rose by 752 on average in individual samples with HGD1 presenting the greatest improvement of 2109.

**Additional file 4.** Table S3. Coefficient of variation (CV%) values of the sums of logarithm base two-transformed LFQ intensities of technical triplicates. The CV% values were calculated in each pancreatic cyst fluid sample (Technical replicate 1: TR1, Technical replicate 2: TR2, Technical replicate 3: TR3).

**Additional file 5.** Table S4. Results of the statistical analysis. Statistical significance, *p* values, and fold-changes for 1751 proteins by Student’s *t* test (Benjamini–Hochberg FDR = 0.05) in three comparison sets (comparison set 1, LGD versus HGD; comparison set 2, HGD versus invasive IPMN; comparison set 3, LGD versus Invasive IPMN).

**Additional file 6.** Table S5. List of exclusively upregulated, exclusively downregulated, and up- or downregulated proteins in all comparison groups. Of the 243 DEPs, 142 were upregulated and 91 were downregulated in at least one comparison group; 10 proteins were up- or downregulated by Student’s *t* test (INV: invasive IPMN).

**Additional file 7.** Table S6. Gene ontology analysis results. GO annotation was performed using the DAVID bioinformatics tool. The *p* value (modified Fisher exact *p* value) cutoff for the GO annotation was set to < 0.05. Genes that were involved in each GO term are provided as official gene symbols. ‘GO FAT’ filters broad GO terms, based on the measured specificity of each term.

**Additional file 8.** Table S7. Demographic and clinical characteristics of the study population for validation. A total of 19 pancreatic cyst fluid samples were used to confirm the credibility of marker candidates.

**Additional file 9.** Table S8. Previous studies that reference the 18 potential biomarker candidates. The associated categories include pancreatic diseases (IPMN, pancreatic cancer, pancreatitis), expression in the pancreas, malignancy, and association with other cancers.

**Additional file 10.** Table S9. Ponceau S intensity. Densitometric analysis of Ponceau S intensities, used as an alternative loading control to actin in western blots (LGD: Low-grade dysplasia, HGD: High-grade dysplasia, INV: invasive IPMN).

## Abbreviations

LGD: low-grade dysplasia
IGD: intermediate-grade dysplasia
HGD: high-grade dysplasia
IPMN: intraductal papillary mucinous neoplasm
MCN: mucinous cystic neoplasm
DEP: differentially expressed protein
MRI: magnetic resonance imaging
CT: computed tomography
CEA: carcinoembryonic antigen
CA19-9: carbohydrate antigen 19-9
KRAS: GTPase Kras
GNAS: guanine nucleotide-binding protein alpha subunit
BCA: bicinchoninic acid assay
SDS: sodium dodecyl sulfate
ABC: ammonium bicarbonate
TFA: trifluoroacetic acid
HPLC: high-performance liquid chromatography
RP: reverse phase
ACN: acetonitrile
HCD: higher-energy collisional dissociation
NCE: normalized collision energy
FDR: false discovery rate
GO: gene ontology
BP: biological process
CC: cellular component
MF: molecular function
TMHMM: transmembrane helices
PVDF: polyvinylidene
BSA: bovine serum albumin
ECL: enhanced chemiluminescence
CV: coefficient of variation
LFQ: label-free quantification
DDA: data-dependent acquisition
PDAC: pancreatic ductal adenocarcinoma
HOOK1: protein HOOK homolog 1
PTPN6: tyrosine–protein phosphatase non-receptor type 6
MUC2: mucin 2
FBN1: fibrillin-1
CLDN18: caludin-18
SERPINA5: plasma serine protease inhibitor
TYMP: tyrosine phosphorylase
TLN1: talin-1
YBX1: nuclease-sensitive element-binding protein 1
TEX12: testis-expressed sequence 12 protein
AKR1B10: aldo-keto reductase family 1 member B10
THY1: Thy-1 membrane glycoprotein
MUC5AC: mucin-5AC
CST6: cystatin-M
WFDC2: WAP four-disulfide domain protein 2
PIK3IP1: phosphoinositide-3-kinase-interacting protein 1
SERPINA4: kallistatin
TFF1: trefoil factor 1
