# Supplementary material for: Quantitative proteomic analysis of pancreatic cyst fluid proteins associated with malignancy in intraductal papillary mucinous neoplasms
Source: Clin Proteomics. 2018 Apr 18;15:17. doi: 10.1186/s12014-018-9193-1 (PMC5907296; doi:10.1186/s12014-018-9193-1)
Supplement: Supplementary file 1 — Additional file 1. Supplementary Figures S1–S9. [file 12014_2018_9193_MOESM1_ESM.docx]

**Quantitative proteomic analysis of pancreatic cyst fluid proteins associated with malignancy in intraductal papillary mucinous neoplasms**

Misol Do^1,#^, Dohyun Han^4,#^, Joseph Injae Wang^2^, Hyunsoo Kim^2^, Wooil Kwon^3^, Youngmin Han^3^, Jin-Young Jang^3,*^, and Youngsoo Kim^1,2,*^

^1^Departments of Biomedical Sciences, ^2^Biomedical Engineering, and ^3^Surgery, Seoul National University College of Medicine, 28 Yeongeon-dong, Seoul, 110-799, Korea; ^4^Proteomics Core Facility, Biomedical Research Institute, Seoul National University Hospital, 101 Daehak-ro, Seoul, Korea

**Supplementary Figures**

**
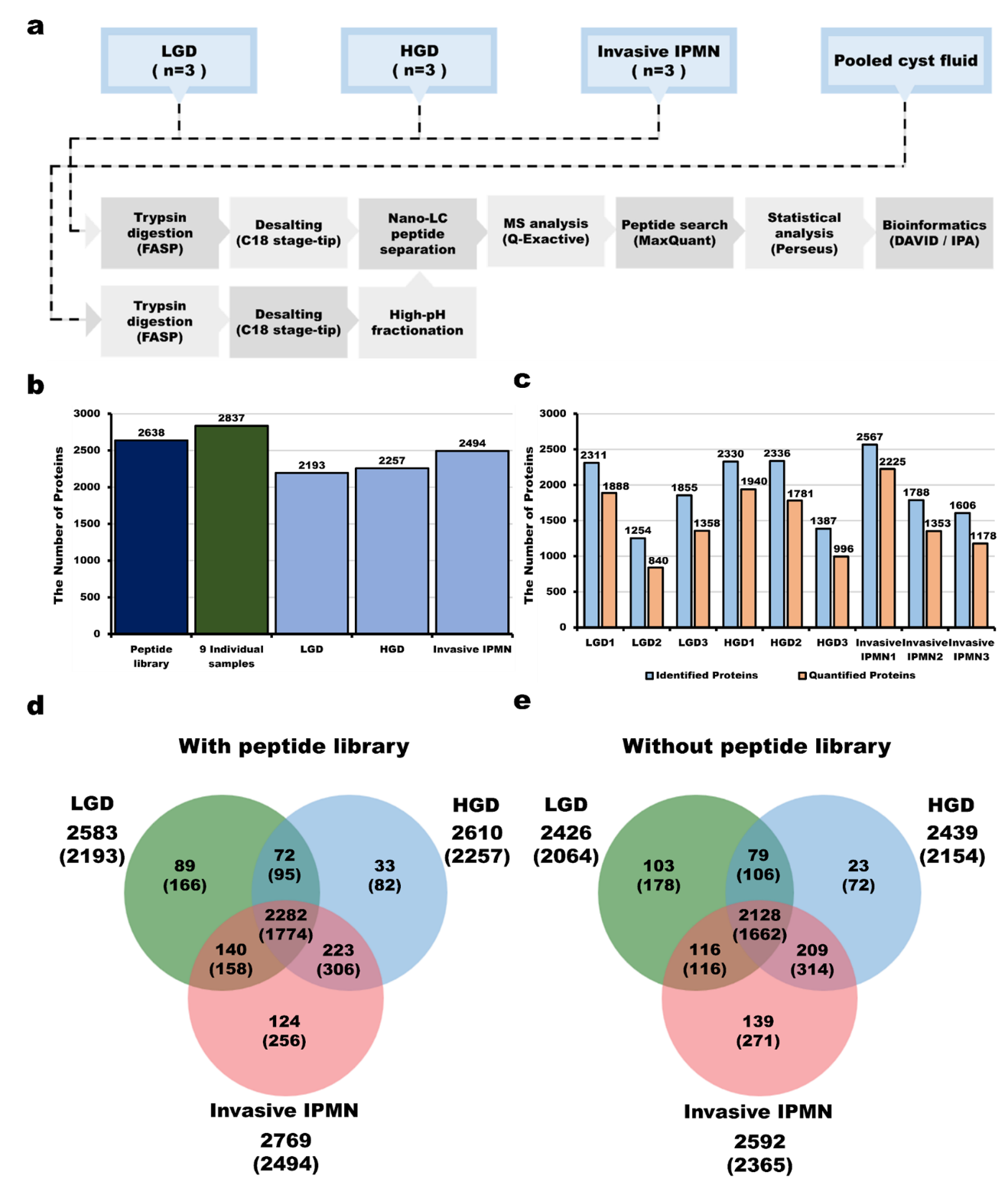
**

**Additional file 1: Figure S1. Experimental workflow of proteomic analysis for IPMN dysplasia and the number of identified and quantified proteins in individual samples and the peptide library. a** Cyst fluids of three types of dysplasia of IPMN and pooled cyst fluid were used for the analysis. The sample preparation and data analysis are shown in the flowchart (LGD, low-grade dysplasia; HGD, high-grade dysplasia; invasive IPMN, invasive intraductal papillary mucinous neoplasm). **b** Quantified proteins in the peptide library, the 9 individual samples, and each histological group of IPMN. **c** Total identified and quantified proteins in each of the 9 individual samples. Identified and quantified proteins in LGD, HGD, and invasive IPMN in the search results, including the peptide library **(d)** and excluding the peptide library **(e)**. The number of quantified proteins is noted in parentheses.

**
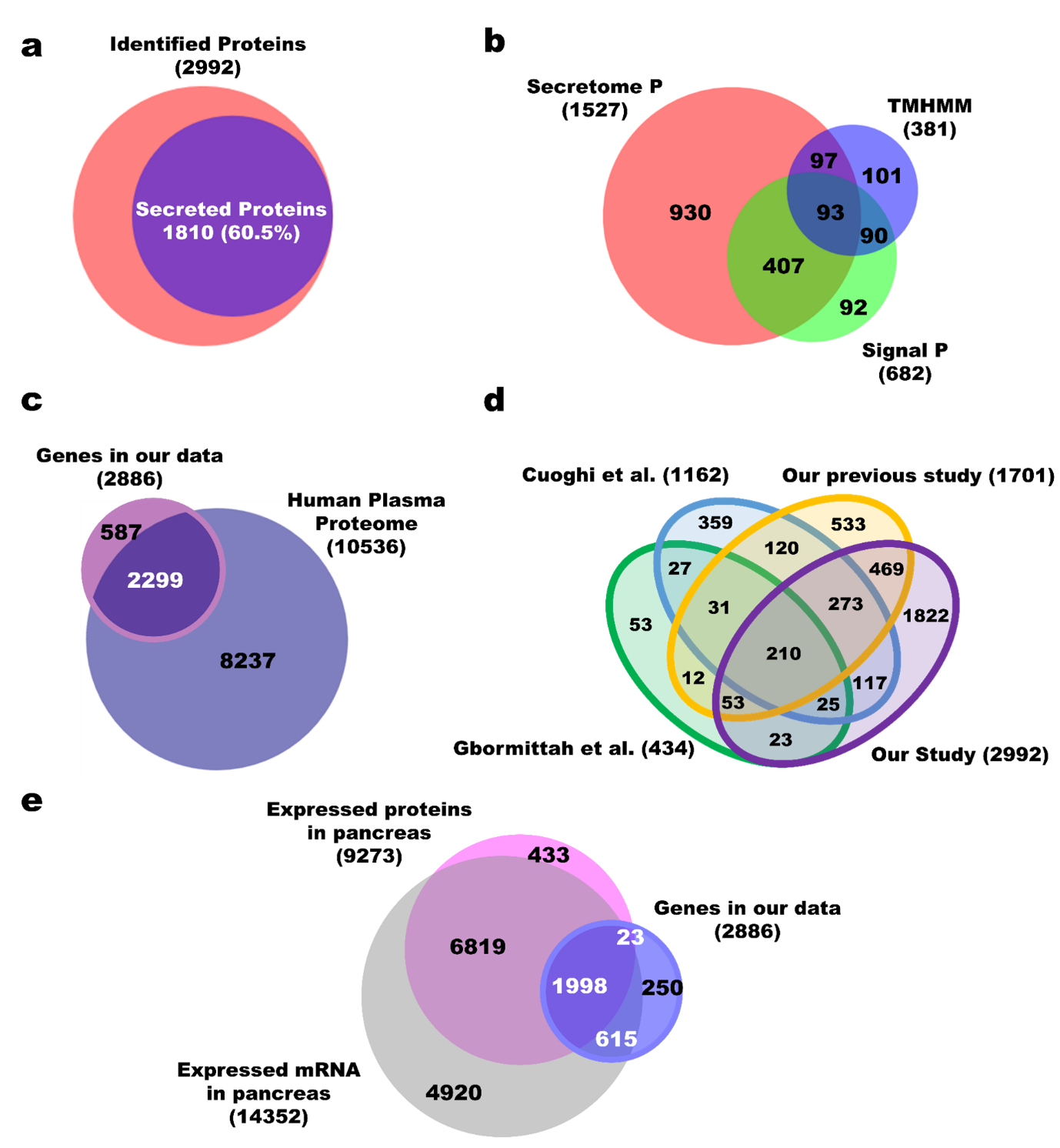
**

**Additional file 1: Figure S2. Comparative analysis with other proteomic databases. a** A total of 1810 of the identified proteins were secreted proteins (60.5%); **b** 1810 secreted proteins were annotated in Secretome P (1527 proteins), Signal P (682 proteins), and TMHMM (381 proteins). **c** Protein accession numbers were converted to gene names, and redundancies were discarded prior to the comparative analysis. Comparing the dataset with the Human Plasma Proteome Database, 2299 (79.7%) of the identified proteins were plasma proteins. **d** Our data had greater depth than those of other proteomics studies on pancreatic cyst fluid (*Cuoghi et al.*, *Gbormittah et al.*) and our previous report. **e** Comparing the dataset of 2886 genes to the Human Protein Atlas, 2613 genes had corresponding mRNA entries and 2021 genes had corresponding protein entries in the pancreas.

**
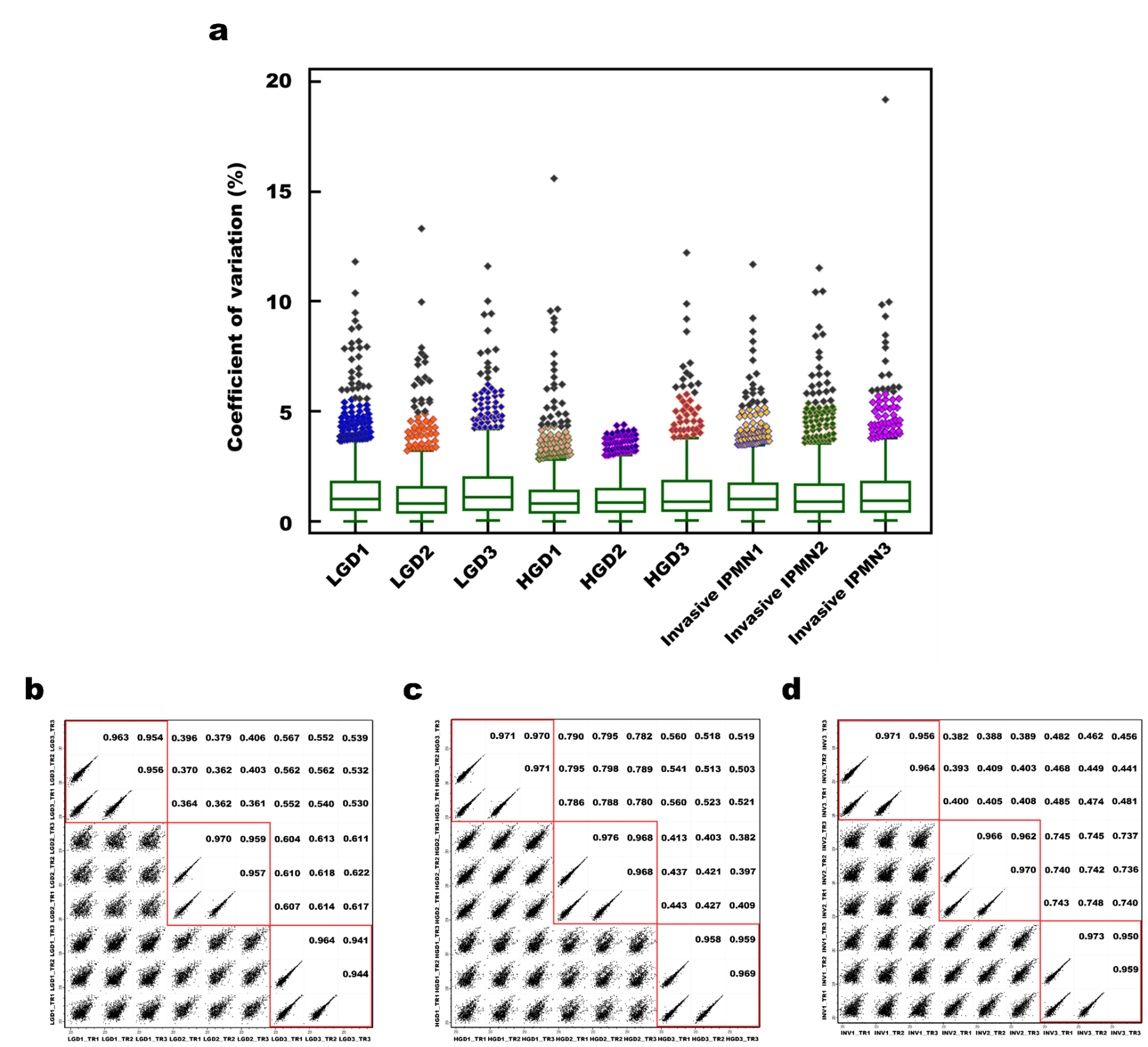
**

**Additional file 1: Figure S3. Coefficient of variation (CV%) values of technical replicates in all individual samples and scatterplot of Pearson correlation coefficients. a** Intensity values of label-free quantification by MaxQuant were transformed to base-2 logarithms, and the coefficient of variation (CV%) values in the technical replicates of the 9 individual cyst fluids were calculated and represented as box plots. Scatterplot of Pearson correlation coefficients of each technical replicate in 3 biological replicates of LGD **(b)**, HGD **(c)**, and invasive IPMN **(d)** (TR1: technical replicate 1, TR2: technical replicate 2, TR3: technical replicate 3, INV : invasive IPMN).

**
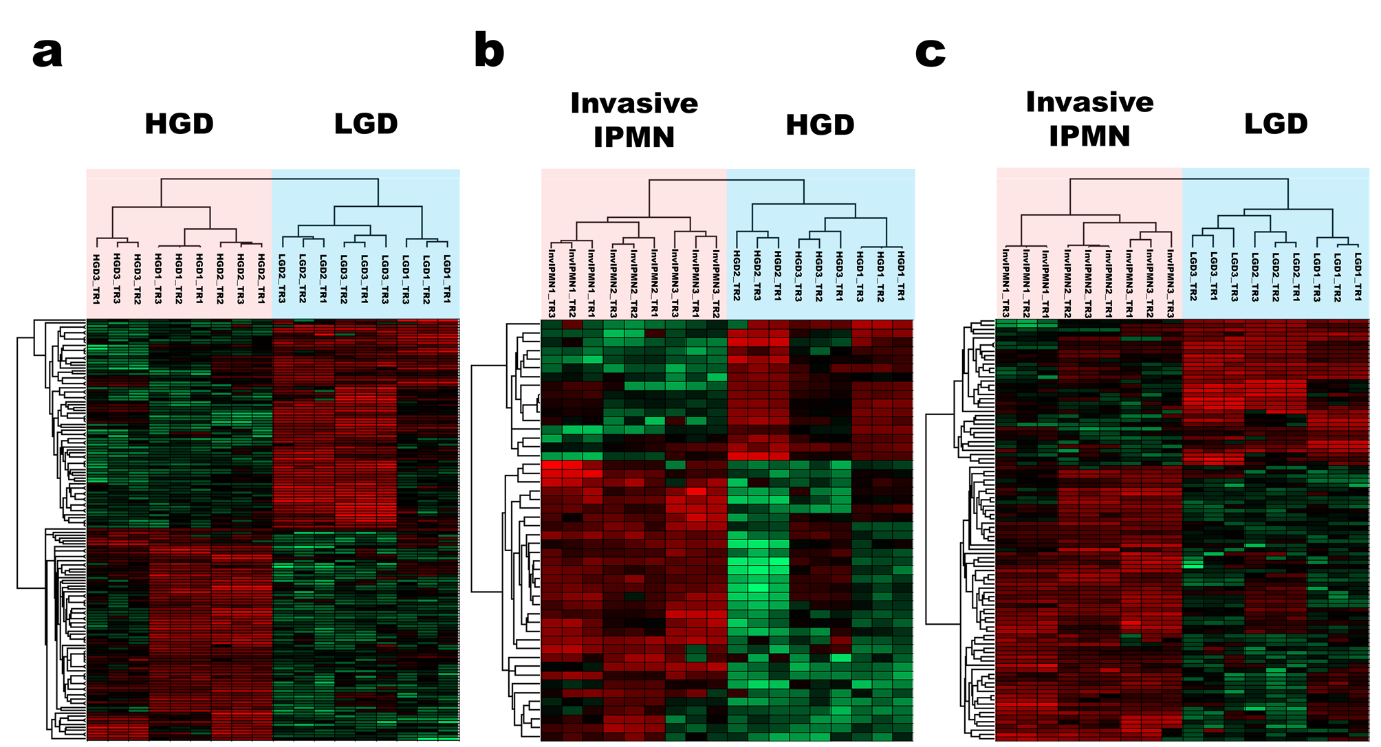
**

**Additional file 1: Figure S4. Hierarchical heat map clusters of significant proteins by student’s t-test.** Hierarchical clustering of differentially expressed proteins after student’s t-test of LGD versus HGD **(a)**, HGD versus invasive IPMN **(b)**, and LGD versus invasive IPMN **(c)**. By student’s t-test, the DEPs clustered in accordance with the histological groups of IPMN.

**
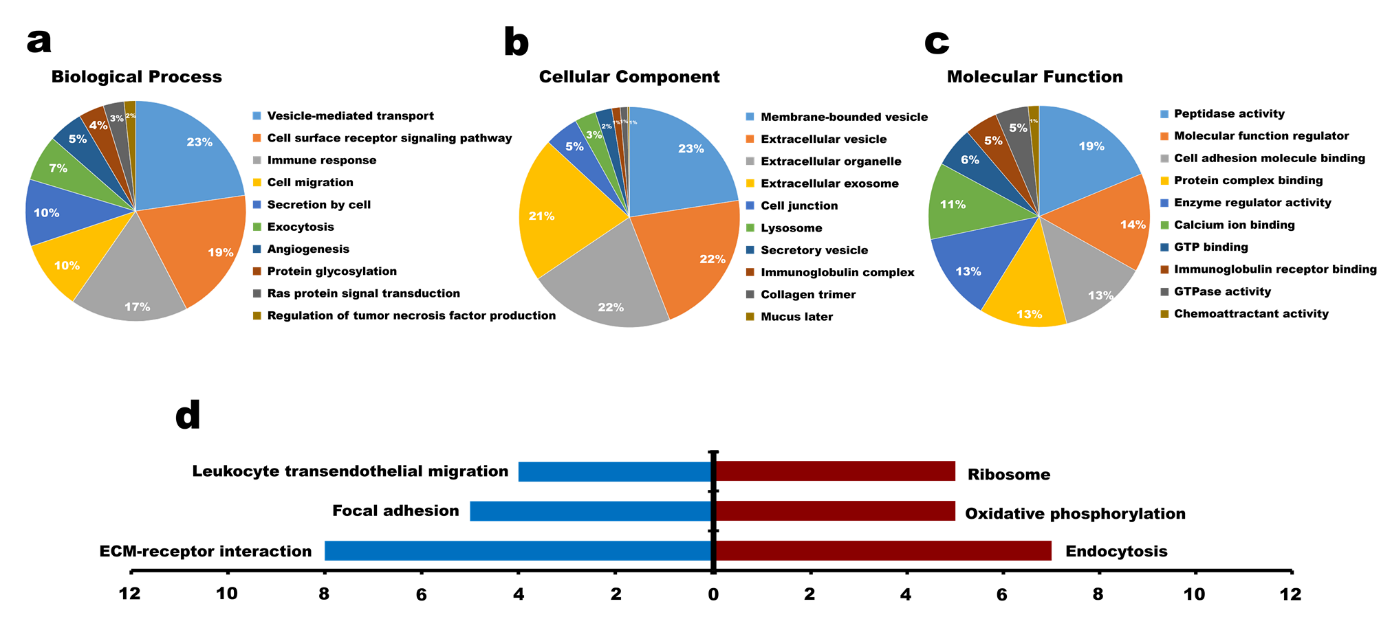
**

**Additional file 1: Figure S5. Gene ontology analysis.** A total of 243 differentially expressed proteins were used in the gene ontology analysis. The percentage of DEPs belonging to **(a)** Biological process, **(b)** Cellular component, and **(c)** Molecular function are shown in pie charts. **(d)** A total of 142 upregulated proteins in all comparison groups were associated with the ribosome, oxidative phosphorylation, and endocytosis; the 91 downregulated proteins in all comparison groups were associated with leukocyte transendothelial migration, focal adhesion, and ECM-receptor interaction.

**
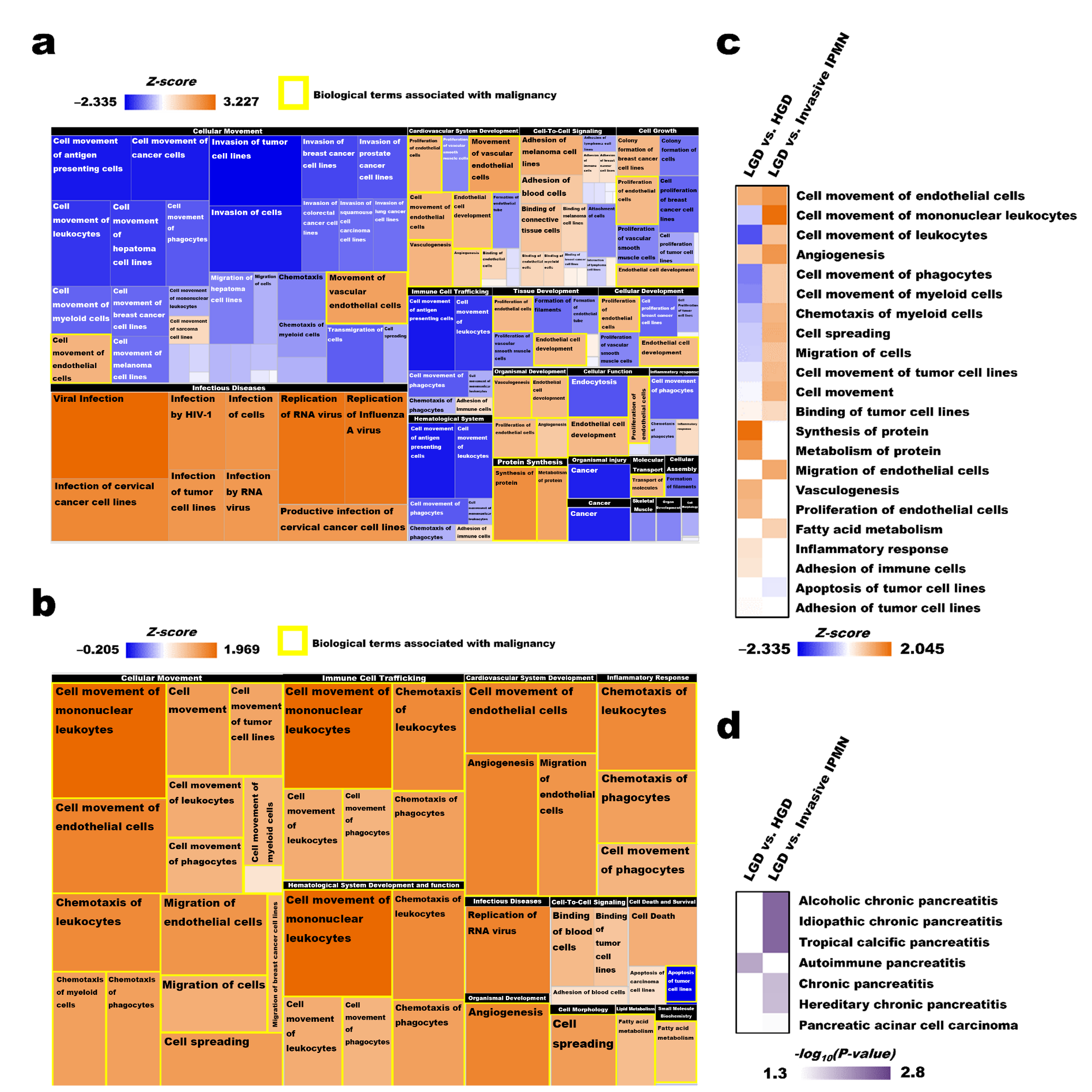
**

**Additional file 1: Figure S6. Ingenuity Pathway Analysis. a** The heat maps represent upregulation and downregulation of biological function based on z-score as squares of various sizes and colors. Larger squares reflect greater z-scores, with orange signifying positive values and blue signifying negative values. When analyzing the 149 DEPs in LGD and HGD, cell-to-cell signaling- and cardiovascular system-related terms were upregulated (-2.335 < z-score < 3.227). The biological terms associated with malignancy are highlighted in yellow. **b** A total of 98 DEPs in LGD and invasive IPMN were upregulated in cellular movement-, immune cell trafficking-, and inflammatory response-related terms. These DEPs were downregulated with regard to apoptosis of tumor cell lines (-0.205 < z-score < 1.969). The biological terms associated with malignancy are highlighted in yellow. **c** Various terms in Diseases and Functions were represented in accordance with the 2 comparison groups. Orange represents a positive z-score, and blue represents a negative z-score (-2.335 < z-score < 2.045). **d** The *p*-values, which represent the correlation between DEPs and pancreatic diseases, are shown in accordance with the 2 comparison groups. Darker colors reflect greater association with the disease (1.3 < -log10 of Fisher’s exact test *p*-value < 2.8).

**
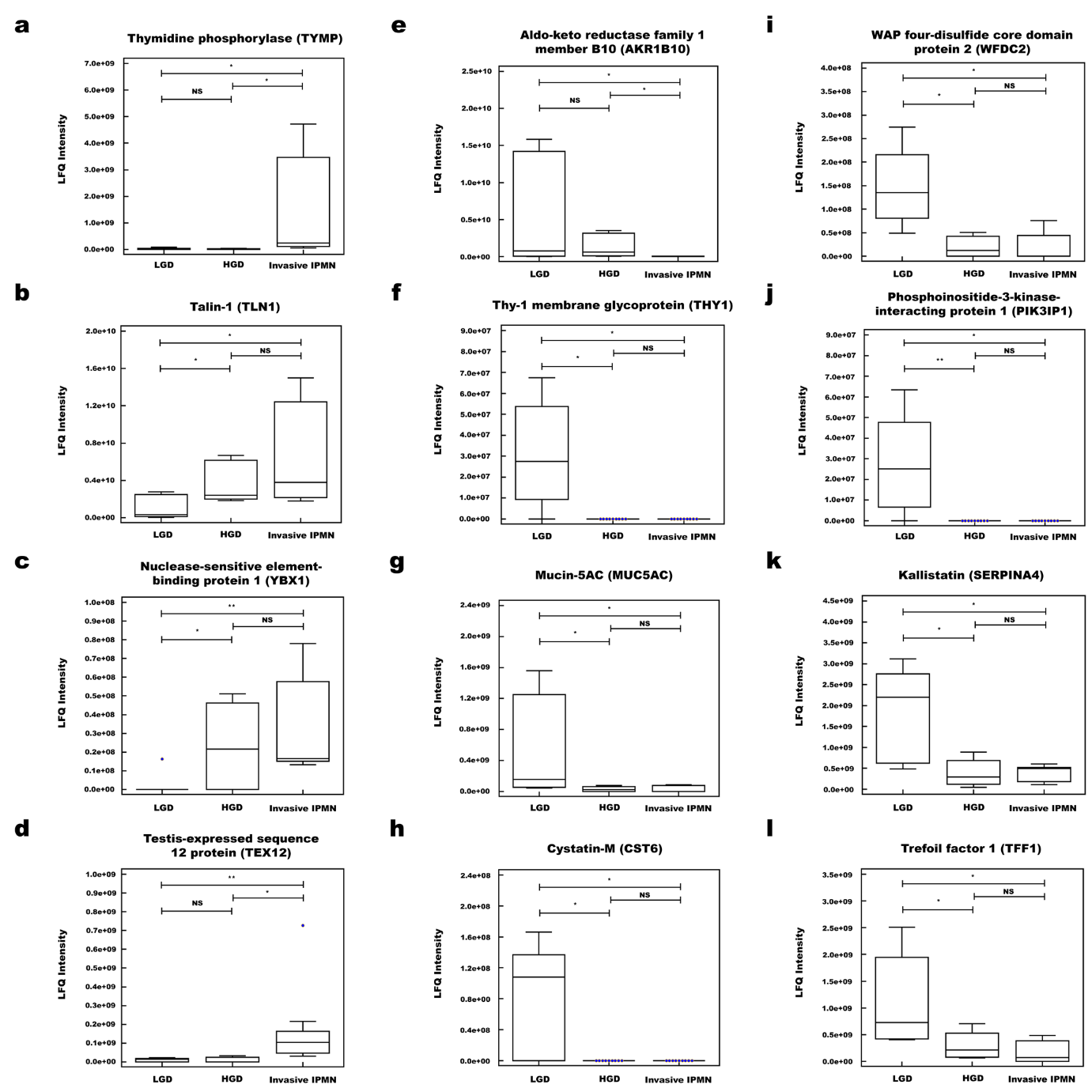
**

**Additional file 1: Figure S7. Twelve potential biomarkers with expression patterns that were consistent with the degree of malignancy. a - d** are predominantly expressed in invasive IPMN. **e - l** are dominantly expressed in LGD (* < p-value 0.05, ** < p-value 0.01, *** < p-value 0.001, **** < p-value 0.0001, NS: not significant).

**
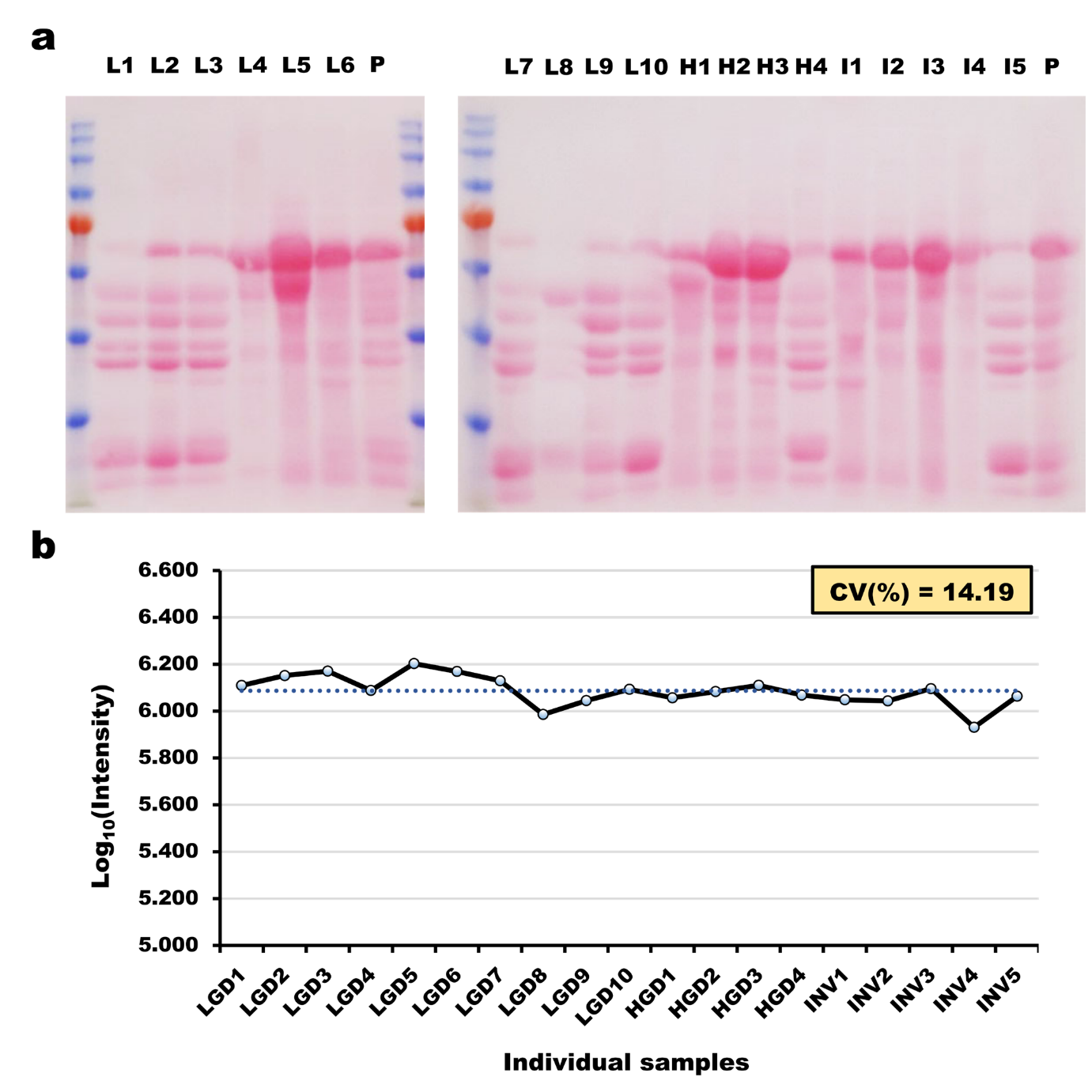
**

**Additional file 1: Figure S8. Ponceau S staining as an alternative loading control to actin. a** Image of Ponceau S staining (L: LGD, H: HGD, I: invasive IPMN, P: Pooled cyst fluid sample). **b** Profile plot representation of base-10 log-transformed Ponceau S intensity values. The average (6.087) is shown as a dotted line, and the coefficient of variation (14.19%) is written above the plot (INV: Invasive IPMN).

**
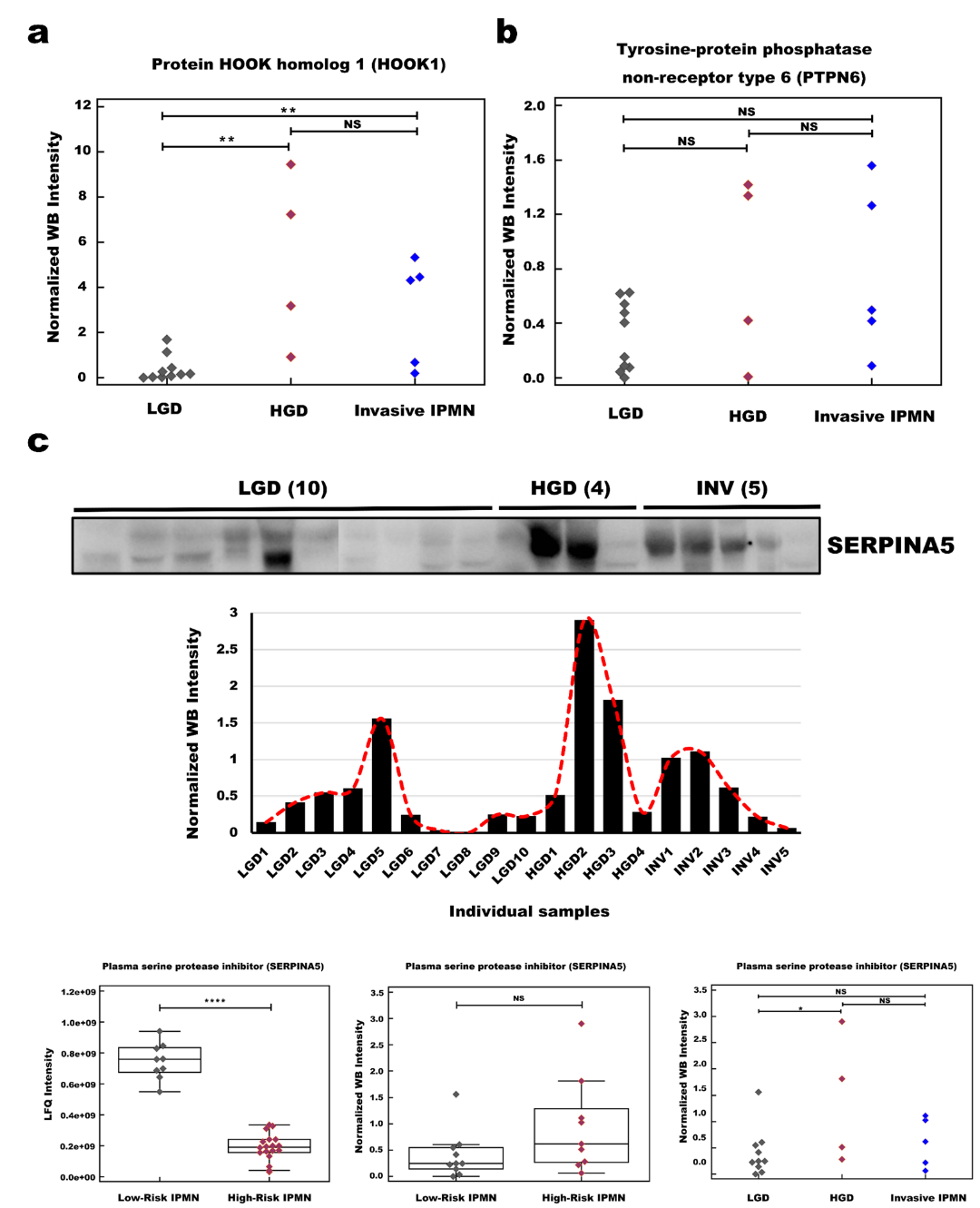
**

**Additional file 1: Figure S9. Validation of HOOK1, PTPN6, and SERPINA5 as potential biomarker targets by western blot. a** HOOK1 was expressed in higher levels in HGD and invasive IPMN than in LGD (p-value <0.01). No significant difference was observed in the expression between HGD and invasive IPMN. **b** No statistically significant difference was observed in the expression pattern of PTPN6 between the 3 comparison groups. The general immunoblotting results are in line with our proteomic results. **c** A total of 19 pancreatic cyst fluid samples were analyzed by western blot to validate the relative abundance of SERPINA5. The immunoblotting results were inconsistent with our MS results. Although SERPINA5 was overexpressed in low-risk IPMN according to LFQ intensity (p-value < 0.0001), the immunoblotting result demonstrated that SERPINA5 expression is higher in HGD than in LGD (p-value < 0.05) (* < p-value 0.05, ** < p-value 0.01, *** < p-value 0.001, **** < p-value 0.0001, NS: not significant, INV: invasive IPMN).

**References**

1. Wisniewski JR, Mann M.: Consecutive proteolytic digestion in an enzyme reactor increases depth of proteomic and phosphoproteomic analysis. Anal Chem 2012, 84:2631-2637.

2. Rappsilber J, Mann M, Ishihama Y.: Protocol for micro-purification, enrichment, pre-fractionation and storage of peptides for proteomics using StageTips. Nat Protoc 2007, 2:1896-1906.
